# Implications of localized charge for human influenza A H1N1 hemagglutinin evolution: Insights from deep mutational scans

**DOI:** 10.1371/journal.pcbi.1007892

**Published:** 2020-06-25

**Authors:** Chadi M. Saad-Roy, Nimalan Arinaminpathy, Ned S. Wingreen, Simon A. Levin, Joshua M. Akey, Bryan T. Grenfell

**Affiliations:** 1 Lewis-Sigler Institute for Integrative Genomics, Princeton University, Princeton, New Jersey, United States of America; 2 MRC Centre for Global Infectious Disease Analysis, Department of Infectious Disease Epidemiology, School of Public Health, Imperial College London, London, United Kingdom; 3 Department of Molecular Biology, Princeton University, Princeton, New Jersey, United States of America; 4 Department of Ecology and Evolutionary Biology, Princeton University, Princeton, New Jersey, United States of America; 5 Woodrow Wilson School of Public and International Affairs, Princeton University, Princeton, New Jersey, United States of America; 6 Division of International Epidemiology and Population Studies, Fogarty International Center, National Institutes of Health, Bethesda, Maryland, United States of America; Emory University, UNITED STATES

## Abstract

Seasonal influenza A viruses of humans evolve rapidly due to strong selection pressures from host immune responses, principally on the hemagglutinin (HA) viral surface protein. Based on mouse transmission experiments, a proposed mechanism for immune evasion consists of increased avidity to host cellular receptors, mediated by electrostatic charge interactions with negatively charged cell surfaces. In support of this, the HA charge of the globally circulating H3N2 has increased over time since its pandemic. However, the same trend was not seen in H1N1 HA sequences. This is counter-intuitive, since immune escape due to increased avidity (due itself to an increase in charge) was determined experimentally. Here, we explore whether patterns of local charge of H1N1 HA can explain this discrepancy and thus further associate electrostatic charge with immune escape and viral evolutionary dynamics. Measures of site-wise functional selection and expected charge computed from deep mutational scan data on an early H1N1 HA yield a striking division of residues into three groups, separated by charge. We then explored evolutionary dynamics of these groups from 1918 to 2008. In particular, one group increases in net charge over time and consists of sites that are evolving the fastest, that are closest to the receptor binding site (RBS), and that are exposed to solvent (i.e., on the surface). By contrast, another group decreases in net charge and consists of sites that are further away from the RBS and evolving slower, but also exposed to solvent. The last group consists of those sites in the HA core, with no change in net charge and that evolve very slowly. Thus, there is a group of residues that follows the same trend as seen for the entire H3N2 HA. It is possible that the H1N1 HA is under other biophysical constraints that result in compensatory decreases in charge elsewhere on the protein. Our results implicate localized charge in HA interactions with host cells, and highlight how deep mutational scan data can inform evolutionary hypotheses.

## Author summary

The hemagglutinin (HA) surface protein of influenza A viruses evolves rapidly to evade host immunity, leading to sizable yearly epidemics in human populations. Previous transmission experiments with H1N1 in mice have tied immune escape to an increase in HA avidity for cellular receptors, mediated by electrostatic charge. Furthermore, retrospective sequence analyses from a previous study confirmed that the HA of circulating global H3N2 has increased in net charge, yet surprisingly, that of H1N1 has not varied significantly. How is a stable net charge related to local patterns of H1N1 HA charge in response to selection? To elucidate the role of local electrostatic charge in host-virus interactions, we investigate characteristics of local charge on the H1N1 HA using functional data from deep mutational scan experiments. Combining measures of functional selection and expected charge at each site on DMS data from a 1933 H1N1 HA yields a striking visual pattern that identifies three groups of sites that have different biophysical properties and that prove to have distinct evolutionary patterns in natural human sequences. Essentially, we find evidence for an increase in charge near the receptor binding site and for compensatory changes elsewhere on the protein. Thus, our findings may reconcile disparate results from transmission experiments and natural sequence analyses, and highlight the importance of local properties of the HA protein. Overall, our findings further support the hypothesis of immune escape due to increased HA avidity to cellular receptors mediated by electrostatic charge. More generally, our work illustrates a novel method of leveraging deep mutational scans in conjunction with natural sequences to shed light on virus-host interactions.

## Introduction

Influenza A viruses (IAVs) are responsible for a major burden of disease in human populations [3]. IAVs are segmented RNA viruses, generally characterized by the two surface glycoproteins hemagglutinin (HA) and neuraminidase (NA) [4]. Much of the acquired humoral host immune response to IAVs acts against the HA, which is responsible for cell entry. The HA consists of two subunits, HA1 and HA2, the former containing the receptor binding site (RBS) and exhibiting greater variation, while the latter is more antigenically conserved. Phylogenetically, HAs of IAVs form two monophyletic clades, known as group 1 and 2, and each group contains multiple subtypes, including the HA of H1N1 and H3N2, respectively [5]. These viruses evolve via two mechanisms. Strong selective pressures from the host immune system, combined with error-prone RNA polymerases, lead to seasonal variants of a given subtype emerging through “drift” [6]. These yearly epidemics contrast to pandemics caused by a “shift” that occurs when IAVs of different animal species reassort, resulting in viruses with antigens to which the human population has little prior immunity [7].

**Fig 1:**
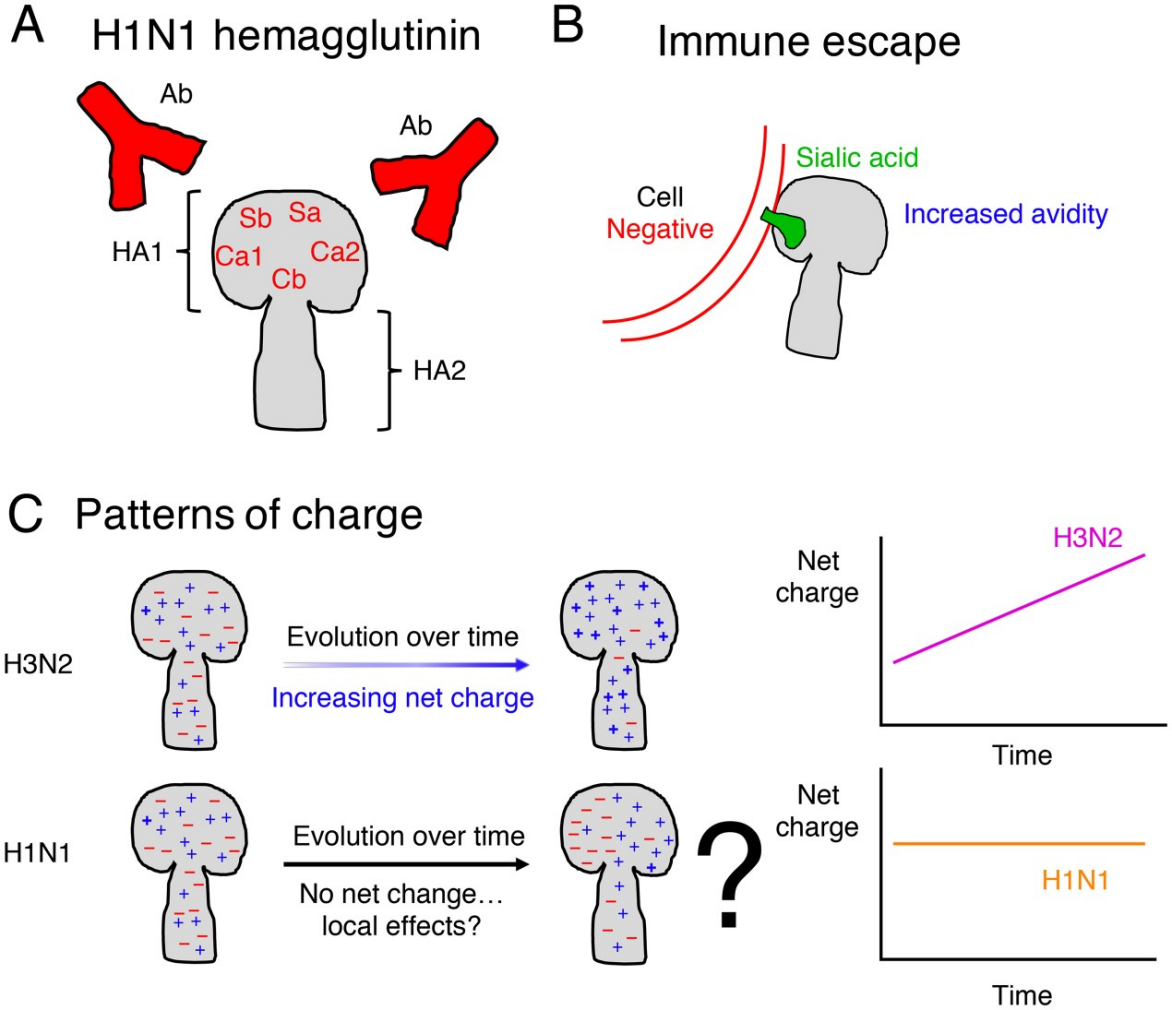
Schematic of host-virus interactions at the hemagglutinin (HA) viral surface protein. (*A*) The structure of the H1N1 HA. The immunodominant HA head (HA1) contains the antigenic sites [8] onto which antibodies bind to neutralize the virus, whereas the HA stalk (HA2) is more conserved. (*B*) Immune escape due to increased avidity mediated by electrostatic charge, according to transmission experiments by Hensley and coauthors [1]. To infect host cells, virions must first successfully enter cells. To accomplish this, the HA protein binds to a cellular receptor, *i.e.* sialic acid, on the surface of the cell. An increase in charge is posited to increase HA avidity and therefore facilitate HA binding, leading to successful immune escape. (*C*) Patterns of charge over time in natural human sequences. Arinaminpathy and Grenfell [2] showed that the H3 increases over time, but that the H1 is constant. (*Inset*) Schematic of the net charge trends over time for the HAs of H1N1 and H3N2 viruses in natural human sequences.

As antigenic drift is driven by selection pressures exerted by previously infected hosts, characterizing HA-antibody interactions is crucial to understand successful immune escape. Caton and coauthors [8] experimentally determined the regions on the H1N1 HA onto which antibodies bind, and grouped these residues into the Sa, Sb, Ca1, Ca2, and Cb antigenic sites (Fig 1A). Individual host immune responses to IAV infections are guided by infection history [9]. A key question is to determine the regions on the HA that are most targeted by antibodies. Hemagglutination inhibition (HI) and enzyme-linked immunosorbent (ELISA) assays can establish an immunodominance hierarchy, indicating which sites are most strongly targeted by host antibodies. With the PR8 H1N1 strain in mice, Angeletti and colleagues [10] found this hierarchy to be Sb (highest), Sa, Cb, Ca2, and Ca1 (lowest). In humans and with the A/Michigan/45/2015 H1N1 strain, Liu and coauthors [11] concluded that Sb was the most immunodominant, followed by Sa, Ca1, Ca2, and Cb; Sb and Sa were significantly immunodominant in contrast to Ca1, Ca2, and Cb. In yet another study, Koel and colleagues [12] used viruses with HA mutants to determine if single amino acid changes could lead to immune escape. These experiments identified sites near the RBS responsible for the strongest reduction in HI titers for H1N1 (before 2009). Furthermore, these results tie to statistical findings with H3N2 data which showed that, in contrast to known epitope designations, two residue-specific covariates were most important to explain dN/dS scores: These factors are a residue’s distance to the RBS, in addition to its relative solvent accessibility which distinguishes surface versus internal residues [13].

With an aim toward disease control and pandemic preparedness, there have been considerable efforts to identify and characterize IAV dynamics across scales from molecular to global by confronting theory with data [14]. However, the role of molecular characteristics such as glycosylation and electrostatic charge in evolutionary dynamics have yet to be fully resolved [15]. Glycosylation is known to be involved in IAV evolution [16], and Kobayashi and Suzuki [17] examined net charge in the context of N-linked glycosylation patterns over time. Mechanistically, electrostatic charge may play an important role in biomolecular interactions between virions, host cells, and antibodies. Local charge could also be involved in HA interactions with host immunity. As the cell surface is negatively charged, an increase in net charge is posited to aid the HA in binding to the cell surface, and thereby lead to successful immune escape due to more efficient viral entry [18]. Hensley and colleagues [1] illustrated the importance of HA avidity to the RBS in immune escape, and tied this to electrostatic charge by examining mutations leading to escape (Fig 1B). Furthermore, the actual number of charged amino acids in H3 was shown to be greatest in the dominant epitope [19]. By considering the five charged amino acids and summing across IAV sequences, Arinaminpathy and Grenfell [2] found that the net charge of the H3N2 HA increased over time (Fig 1C). However, no such increase was found for the H1N1 HA [2] (Fig 1C). In addition to starkly contrasting with the results from natural sequence analyses with H3N2 HAs, these findings are particularly puzzling since the transmission experiments in mice by Hensley and coauthors [1] were with H1N1 viruses. Arinaminpathy and Grenfell [2] proposed other explanations to account for their findings: in particular, they highlighted the possibility that the observed trends are covariates of other, more important factors, or that immune escape could also be the result of other mechanisms.

Could patterns of localized electrostatic charge within the HA reconcile these findings? Perhaps the simplest explanation for the discrepancy between H1N1 and H3N2 HA net charge over time is that the H1N1 HA could be less tolerant to an overly positive net charge, and thus an increase in charge must be accompanied by a decrease elsewhere to keep the net charge relatively constant and preserve proper protein folding and function (more generally, for a discussion of charge and protein stability, see, e.g. [20]). Here, “function” refers to cell entry and viral replication in the absence of immune selection. Therefore, this hypothesis would posit that while certain regions are under strong immune selection to become more positive (in agreement with the immune escape model of Hensley and coauthors [1]), an additional selective pressure acts elsewhere to keep the net H1N1 HA charge constant (thus reflecting the pattern observed by Arinaminpathy and Grenfell [2]). To investigate this hypothesis, local patterns in the HA charge must be examined. Yet, selection for immune escape may modulate residues independent of charge due to function. Thus, while it would be possible to simply group residues based on individual distance to the receptor binding site and to examine their local charge, such an approach would not appropriately account for the functional constraints (on individual residues) that were present from early on in H1N1 HA evolution. Here, we aim to separate local changes in charge due to protein function versus evolutionary pressures from host immune systems, and this requires estimates of local functional selection on the HA protein.

Recent developments in sequencing technologies have led to data-rich experimental studies examining protein evolution. To uncover the fitness landscapes of HA proteins in the absence of immune selection, deep mutational scans (*e.g.*, Araya and colleagues [21], Doud and Bloom [22]) can interrogate HA functional constraints in a site-wise fashion, yielding probability distributions for residue preference at each site, *e.g.*, for the HA of a particular H1N1 strain. Essentially, all possible ∼10,000 point-wise mutations of the HA protein of a particular background strain are constructed, and viruses with these HAs are then passaged in MDCK-SIAT1 cells at low multiplicity of infection [22]. Subsequently, the resulting samples are sequenced, and those viruses that were capable of cell entry and replication are over-enriched, whereas others that were not are depleted. Essentially, these data reveal mutational constraints of HA in a specific background strain, and can aid in discovering regions that are conserved in natural sequences due to viral function, instead of apparent conservation simply due to evolutionary history.

*What is the role of localized electrostatic charge on the evolutionary dynamics of H1N1 HA? Furthermore, can the characteristics of local charge explain the lack of increase in the HA net charge and thus give further evidence for immune escape due to increased HA avidity to cellular receptors?* In this paper, we make use of deep mutational scan data on H1N1 1933/WSN to identify three groups of sites on the HA protein of H1N1: this classification reflects functional constraints (*i.e.*, purifying selection) in conjunction with the expected charge at each residue. While functional constraints on the H1N1 HA may shift over time, we identify groups of residues based on measures of per-site functional selection and expected charge on an early H1N1 strain, and examine how the charge of these groups has changed over time. We show that in natural sequences from 1918 to 2008, these three groups present different trends of net charge, with the notable finding that the group that is generally closer to the RBS, more accessible to solvent, and evolves the fastest, is increasing in charge. These results possibly implicate a role for the net charge of specific regions on the HA of H1N1 IAVs in evolutionary dynamics, particularly in the context of viral binding to the host cell surface.

## Results

### Functional HA branches and net charge evolution

**Fig 2:**
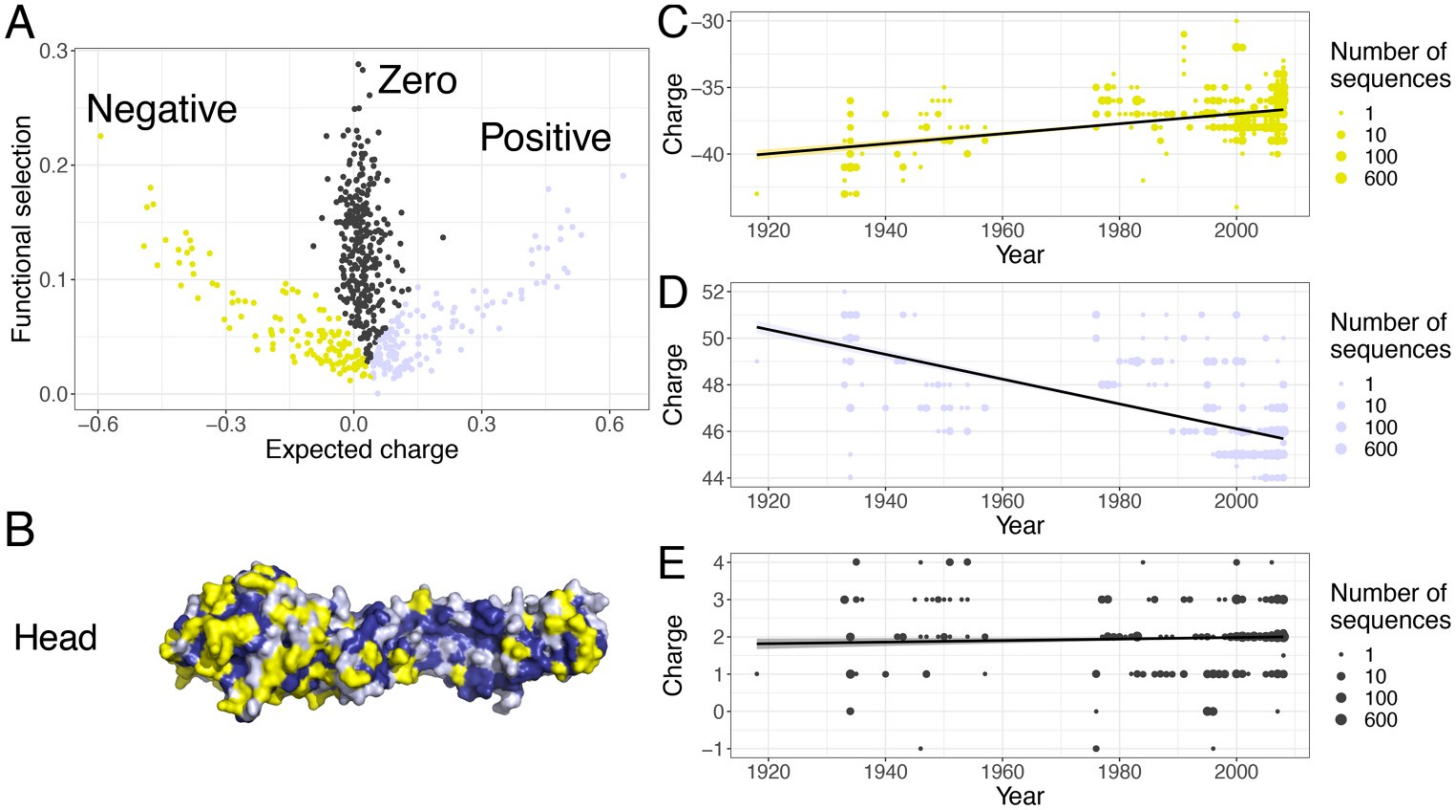
Identification of negative, zero, and positive functional branches. (*A*) Scatter-plot of site-wise functional selection and expected charge, based on deep mutational scanning (DMS). For a given residue using DMS preference data, ‘functional selection’ is a measure of functional constraints at this site (a transformation of Shannon’s evenness) and ‘expected charge’ is the mean charge from DMS preferences. The colours are to facilitate the identification of the negative (yellow), zero (dark blue), and positive (light blue) branches. (*B*) The location of the residues belonging to each of the three branches identified in *A*, on the HA monomer, visualized with PyMOL [24] and PDB 1RVX [23]. *(C)-(E)* The net charge of the positive (*C*), negative (*D*), and zero (*E*) branches over time in 1741 H1N1 sequences from human hosts during 1918 to 2008.

**Fig 3:**
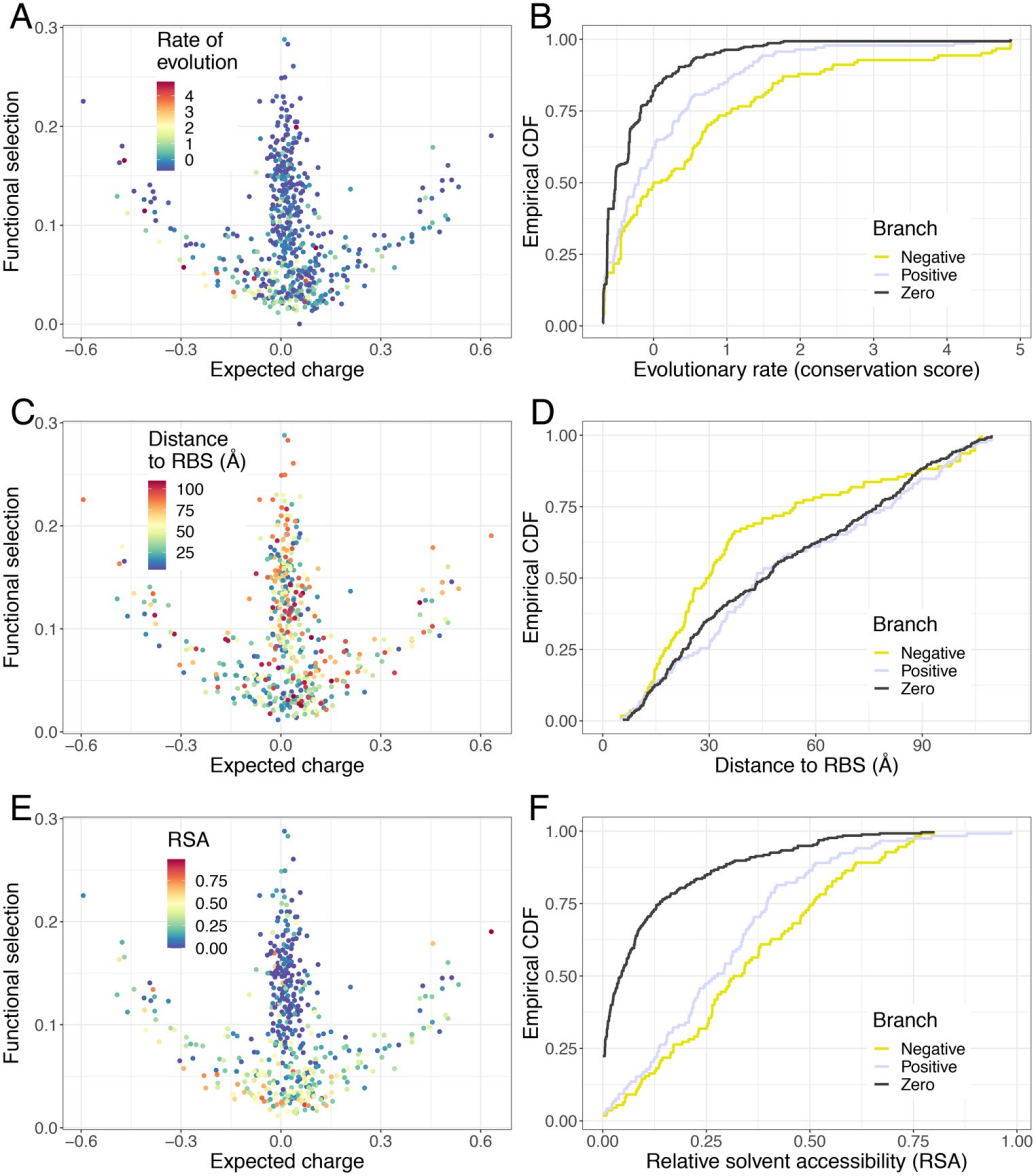
Evolutionary rates, relative solvent accessibility (RSA), and distances to receptor binding site (RBS) on functional branches. (*A*) Normalized conservation scores (labelled rate of evolution) per site overlaid on the functional selection-expected charge plot. These conservation scores were obtained from Rate4site [27] with H1N1 sequences from the fludb database of the IRD [28]. A higher score indicates lower conservation and faster evolution. (*B*) Empirical cumulative distributions of conservation scores for each of the three branches. (*C*) Distances to the RBS overlaid on the functional selection-expected charge plot. These distances were calculated using PyMOL [23] (see Materials and methods) (*D*) Empirical cumulative distributions of the distance to the RBS for each of the three branches. (*E*) Relative solvent accessibility overlaid on the functional selection-expected charge plot. These scores were computed using dmstools2 [29] (see Materials and methods) (*F*) Empirical cumulative distributions of relative solvent accessibility for each of the three branches.

Fig 2 explores how functional selection and expected charge are related, and explores whether these measures can identify distinct groups of residues. By examining the relationship between functional selection and expected charge across the whole protein (Materials and methods), we identified three groups of sites (Fig 2A and 2B). The first set, hereafter named the “negative branch”, exhibits a negative linear relationship between expected charge and functional selection (yellow in Fig 2A). The second set, hereafter named the “positive branch”, mirrors the negative branch by having a positive linear relationship between expected charge and functional selection (light blue in Fig 2A). The last set, hereafter named the “zero branch”, has functional selection independent of expected charge which is clustered around zero charge (dark blue in Fig 2A). Thus, we have now grouped residues on the HA protein based on their functional constraints and charge implications, and Fig 2 illustrates that the distribution of these groups are elaborate. These groupings thus serve as the basis for our further investigations into other properties of the residues in each group (*e.g.* Fig 3).

Having identified these groups, we then explored how the charges of these branches individually evolve over time. In particular, are there any distinct evolutionary patterns that could signal pressures exerted by host immune systems? Using available H1N1 sequences, we computed the net charge of these groups and examined their patterns over time (Materials and methods). From 1918 to 2008, the net charge of the negative branch has significantly increased over time (*P* < 2.2 × 10^−16^). In contrast, the net charge of the positive branch has significantly decreased over time (*P* < 2.2 × 10^−16^). The charge of zero branch does not appear to be changing substantially, though the very slight increase is significant (*P* = 0.0162) (Fig 2C). This positive trend in the zero branch is likely due to two factors that are probably heavily tilting the linear regressions. First, there is an abundance of sequences in certain years compared to others, and second, the net charge on the zero branch is very close to zero and thus heavily influenced by single charged residues. In an effort to remove this source of bias, we performed the same analyses with yearly means, *i.e.*, net charge averaged for each year, and we found that the change in charge of the zero branch was not significant with yearly means (*P* = 0.0976) (S1C Fig). Furthermore, we obtained analogous results for the positive and negative branches with yearly means (S1A and S1B Fig). Therefore, the evolution of net charge on the functional branches exhibits stark differences.

**Fig 4:**
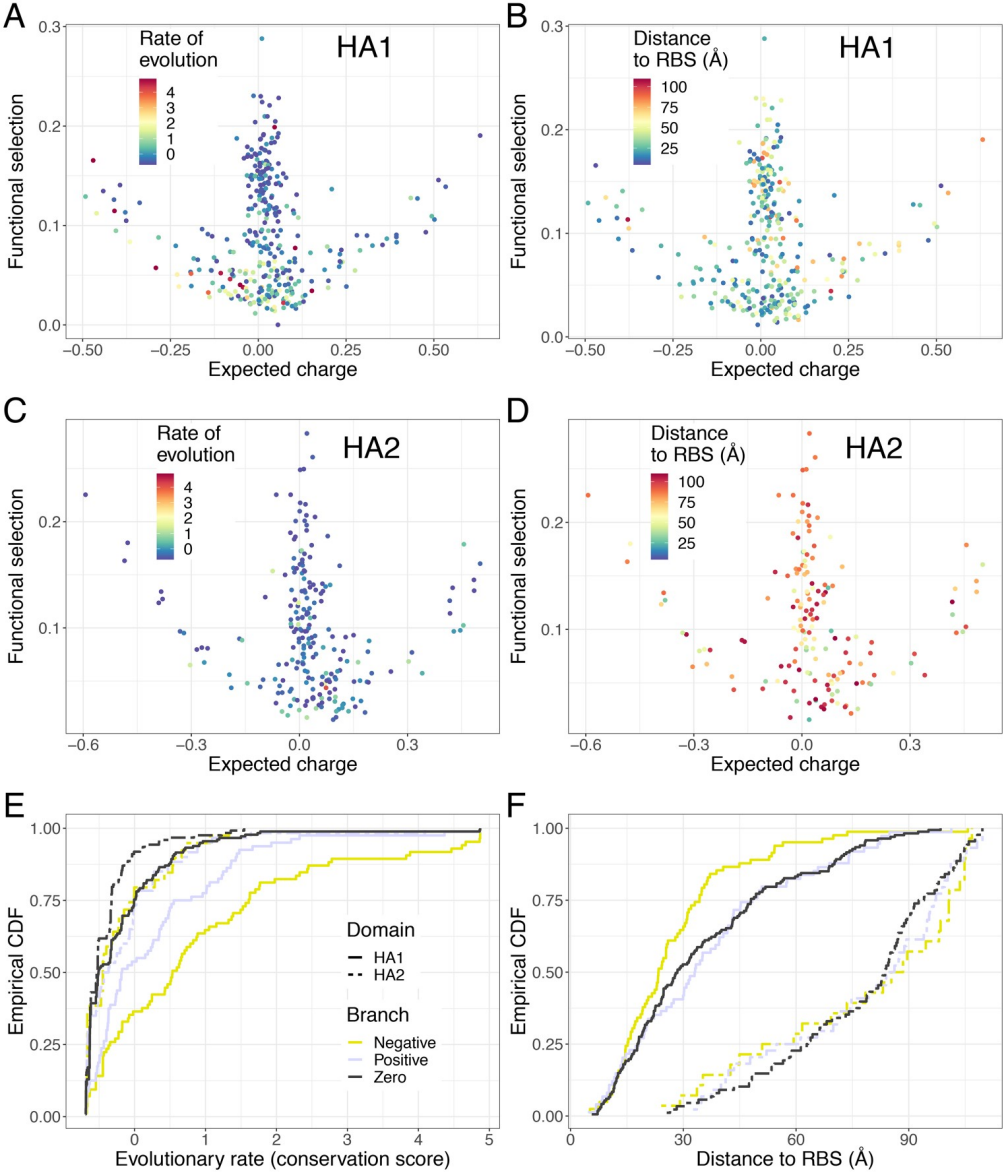
Characteristics of conservation and distance to RBS in each HA subunit. (*A*) Conservation scores (labelled rate of evolution) per HA subunit, overlaid on the functional selection-expected charge plot. These scores were obtained from the Rate4Site algorithm [27] using H1N1 sequences obtained from the IRD fludb database [28] (see Materials and methods). As in Fig 3A, note that higher scores indicate higher rate of evolution. (*B*) Distances to the RBS per HA subunit, overlaid on the functional selection-expected charge plot. These distances were calculated using RBS annotations according to Gamblin and colleagues [24] using PyMOL [23] (see Materials and methods). (*C) and (D) are as in (A) and (B), respectively, but for the HA2 subunit.* (*E*) Empirical cumulative distribution of evolutionary rates of each branch separated by domain. (*F*) Empirical cumulative distribution of distance to RBS, separated by both branch and domain.

The sequence of the HA1 “head” subunit of the HA varies more than the conserved HA2 “stem”. In fact, the HA head is immunodominant and under stronger natural selection due to host immune pressures [25, 26]. Examining per residue functional selection and expected charge for sites in each HA1 and HA2 reveal similar patterns, with HA1 exhibiting more pronounced division into functional branches (Fig 4A and 4C). Thus, we investigated the analogous patterns for these functional branches on the immunodominant HA head. Restricting our analysis to sites that belong to the HA1 subunit, the net charges of the negative and positive branches have significantly increased and decreased over time, respectively (S2A and S2B Fig), just as when considering all HA residues. Also, a linear regression reveals a significant increase in the net charge over time for the HA1 sites in the zero branch, which is clearly increasing from a net charge of about 0 to about 2 (S2C Fig). As before, we accounted for factors that are possibly affecting the regression by averaging sequences within each year. For these yearly means, the net charge of the HA1 zero branch does not vary over time (S3C Fig), whereas those for the HA1 negative and positive branches have significant trends matching our previous results (S3A and S3B Fig). As our sequences date from 1918 (compared to 1977 in Arinaminpathy and Grenfell [2]), we verified that there is no positive trend in net charge over time (S4A Fig) and in fact found a slightly negative trend, but this trend is likely due to uneven sampling over time as it disappeared once yearly means were considered (S4B Fig).

### Evolutionary rates along functional branches

Rates of evolution, quantified as Rate4Site conservation scores, clearly show that the negative branch is evolving faster than the positive branch (Fig 3A and 3B, where a higher score means a higher rate of evolution). Note that these scores are normalized across the whole protein, giving a mean of zero and standard deviation of one. Pairwise Kolmogorov-Smirnov tests reveal that the distributions of conservation scores among the three branches are all statistically different (*D* = 0.21221 and *P* = 0.005356, *D* = 0.27625 and *P* = 9.274 × 10^−7^, *D* = 0.36237 and *P* = 1.926 × 10^−10^ for comparisons between negative-positive, positive-zero, and negative-zero branches, respectively). Moreover, most of these rapidly evolving sites in the negative branch are in the HA1 subunit (Fig 4A) instead of the HA2 subunit (Fig 4C). Analyses with only HA1 sites reveal the same patterns (Fig 4E).

These functional branches we identified may be related to other biochemical properties of HA residues. Meyer and Wilke [13] used statistical models of H3N2 HA data to show that distances to the RBS and relative solvent accessibilities (RSAs) of residues explained a significant fraction of the variance seen in evolutionary rates. On a coarse scale, our H1N1 HA analyses largely agree with these findings. With respect to distances of each residue’s centroid to the centroid of the RBS, the negative branch has a significantly different distribution than either the positive or the zero branch (*D* = 0.28228 and *P* = 0.0001638, *D* = 0.24011 and *P* = 0.000219, respectively), but the zero and positive branch are not significantly different (*D* = 0.10263 and *P* = 0.3347) (Fig 3C and 3D), and these results also hold when only HA1 sites are considered (Fig 4B and 4F). For RSAs, the distribution of the zero branch differs significantly from the other two (*D* = 0.5672 for negative branch and *D* = 0.52792 for positive branch, giving *P* < 2.2 × 10^−16^ in both cases), and the distributions of the positive and negative branches are also significantly different (Fig 3E and 4F, *D* = 0.18629 and *P* = 0.03846). These results are largely the same for analogous comparisons with sites on HA1 (Fig 4, S5 Fig). Furthermore, rates of evolution and distances to RBS generally have greater ranges for residues in HA1 than for those in HA2 (Fig 4).

In summary, the sites that are on the negative branch evolve faster, are generally closer to the RBS, and are on the protein surface. The sites on the positive branch are generally further away from the RBS, but are also on the protein surface. Lastly, the sites on the zero branch are further away from the RBS, and are more likely to be internal residues. As a form of control to our study, we performed the same analyses with TEM-1 beta-lactamase (*i.e.*, not under similar immune selection), to see if the three branches due to functional selection and expected charge are conserved on such a different protein. Importantly, the underlying shape of the data mapped on the functional selection and expected charge axes is quite different from that of the H1N1 HA. Indeed, beta-lactamase appears to possess a very different pattern from that of H1N1 HA, with a clear absence of positive or negative branches. Through further visual inspection, we found no patterns for the distributions of RSA or evolutionary rates as a function of functional selection and expected charge (S6 Fig).

### Local charge distribution

**Fig 5:**
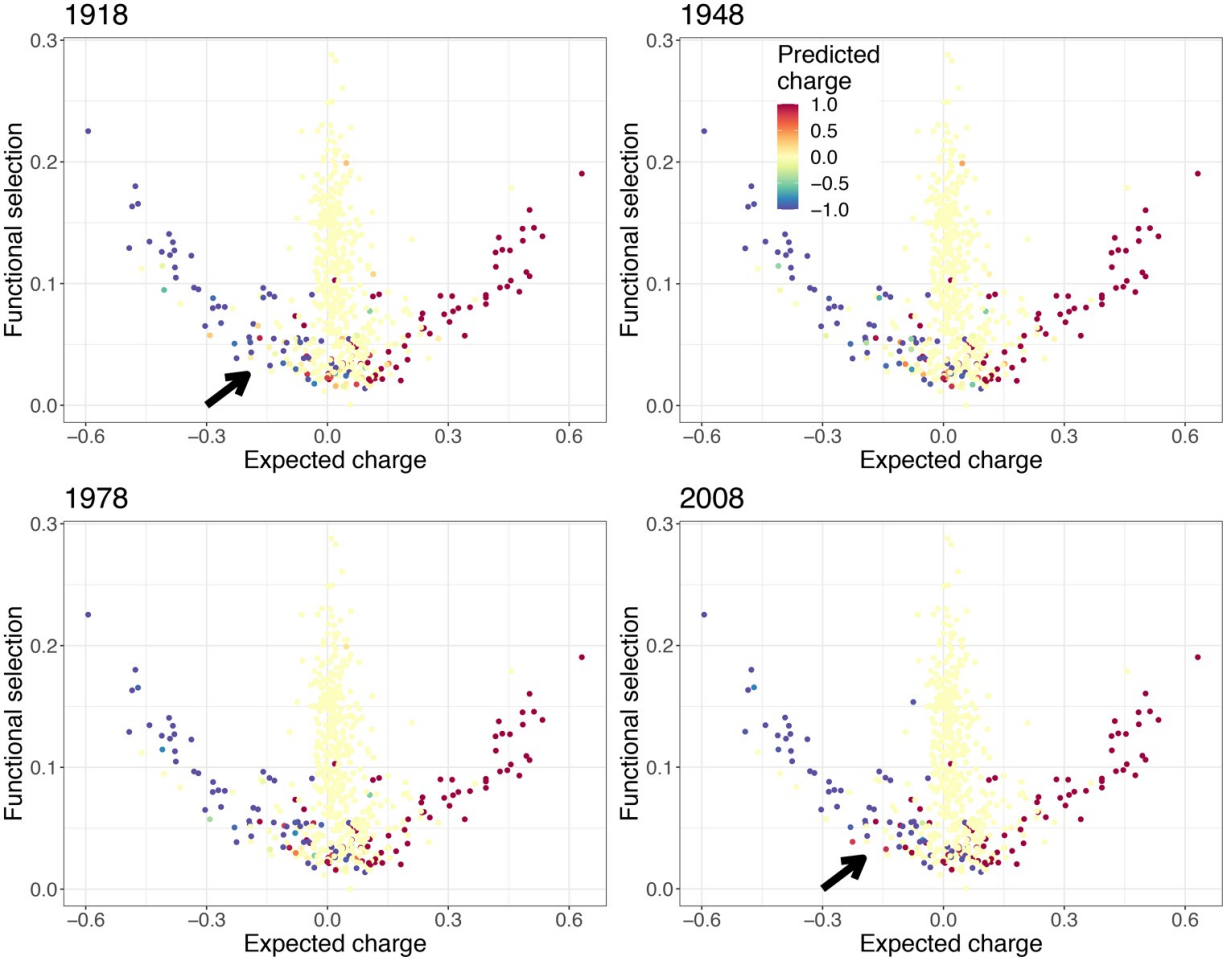
Fitted observed charge in 1918, 1948, 1978, and 2008 computed from fitted per-site multinomial logistic regression models. These models were fitted using nnet [30] in R [31] (see Materials and methods). All 1744 human HA H1N1 sequences between 1918 and 2008 from fludb [28] were used to fit these statistical models. The arrow is used to highlight sites in the negative branch that are transitioning to positive values.

The previous results on the different patterns of net charge for the identified branches have relied upon the aggregation, through summing, of the charges of all sites in each branch. While the net charge on these branches have distinct patterns over time, individual charges per site over time may themselves reveal notable features. To explore this, we fit multinomial logistic regression models at each site in the HA protein to obtain predicted probabilities of given charges over time, using collected natural sequences of H1N1 in humans (Materials and methods). The computed fitted observed charge over time at each site shows that the zero branch remains largely neutral (Fig 5). Moreover, as expected, predicted observed charges on the negative and positive branches tend to be negative and positive, respectively (Fig 5). However, over time, while certain sites in the negative branch do tend to become more negative as expected due to their functional constraints, others appear to transition away from a negative charge (see arrows in Fig 5). This results in the increase in net charge seen for the negative branch (Fig 2C). A similar, but opposite, process appears to occur on the positive branch, where certain residues transition away from a positive charge.

We then investigated how other immunologically relevant biochemical properties such as glycosylation and receptor binding avidity tie in to our results. As glycosylation has been implicated in IAV dynamics (*e.g.* Altman and colleagues [16]), we computed the probabilities of glycosylation at each site from the deep mutational scan data assuming site independence (Materials and methods), and no striking patterns emerged with respect to their distributions amongst the 565 sites (S7 Fig). The assumption of site independence follows from DMS experiments which study functional fitness of single amino acid variants on a specific background strain. To relax this assumption, DMS data examining covariation of multiple residues would be required. Yet, glycosylation for the HA H1N1 is less pronounced than for H3N2 [16], and perhaps plays a less substantial role in H1N1 HA evolution. Hensley and coauthors [1] found certain sites that influence binding by changing the HA avidity to the receptor binding site. Examining the locations of these sites on the functional selection-expected charge plot, it is clear that they are found preferentially on the zero and negative branches (S8 Fig).

### Testing cluster distributions with hierarchical clustering

So far, our results have relied upon the designations of residues belonging to the negative, positive, and zero branches through visual inspection generally supported by RSA values, and this method imposes a hard separation of negative and positive sites near the axis of symmetry. It is important to test if our results are robust to another, more formal, clustering scheme. To further investigate this, we used hierarchical clustering in R [31]. Using unscaled selection coefficients and expected charge values at each residue, we used Ward’s method [32] in R [31] but with Euclidean distance (between residues on the selection–charge plane) instead of its square as a distance measure. Among all possibilities, we used this clustering method since it accurately discriminates the three branches that are visually striking. Murtagh and Legendre [33] show that this distance metric is distinct from that described by Ward [32], but nevertheless it is better at detecting the three branches here, and so we used it to investigate the characteristics on the branches and to show robustness of our results with an alternative to visual grouping. The separation into the three branches is largely consistent with our initial classification. However, the positive branch contains most of the sites that are at the base of the three branches (S9 Fig). Detailed analyses are shown in S1 Appendix, and S9, S10 and S11 Figs. In essence, we found a similar clustering to our more subjective methods. There are subtle differences, but these do not affect the qualitative results.

## Discussion

Transmission studies [1] and natural sequence analyses [2] for H1N1 HA give seemingly contrasting evidence on the role of electrostatic charge on IAV evolution. Indeed, Hensley and colleagues [1] demonstrated that successful immune escape was driven by increased HA H1N1 avidity and mediated by increases in electrostatic charge. Yet, Arinaminpathy and Grenfell [2] found that the H3N2 HA has increased in net charge since 1968, whereas the H1N1 HA has had no appreciable change. To reconcile the very different H1N1 and H3N2 charge trajectories, we investigated the hypothesis that local charge is more germane than global charge for evolutionary dynamics. Moreover, this hypothesis would imply that differences in global patterns of H1N1 and H3N2 HA simply reflect disparities in net charge constraints, instead of contrasting evolutionary pressures. Local charge in natural sequences reflects a combination of functional constraints and evolutionary pressures from host immunity. To isolate these confounding factors and remove bias due to functional constraints, partitioning of residues into functional patches requires per-residue selection experiments in the absence of immune selection on an early H1N1 strain. Constraints of functional relevance for the H1N1 HA are quantified in DMS data for an H1N1 HA from 1933 [22]. By leveraging these data, we found patches on the HA with distinct evolutionary patterns in natural sequences. It should be noted that, due to evolution, similar functional experiments with more recent H1N1 HAs could reveal different groups of residues. However, our analyses require an early H1N1 HA to properly characterize the starting landscape and enable us to appropriately determine the net charge patterns of these patches afterwards.

What are the characteristics of localized electrostatic charge in the H1N1 HA protein evolutionary patterns? Through measures of site-wise functional selection and expected charge obtained from DMS data, we identified three distinct branches of residues. On these “negative”, “positive”, and “zero” branches, functional selection and expected charge are positively correlated, negatively correlated, and independent of each other, respectively. The negative branch consists of residues that are the closest to the RBS, have high RSAs, and evolve the fastest. The positive branch has residues that are similarly surface exposed, but are further from the RBS and evolve slower. Lastly, the residues in the zero branch are those that are buried in the core of the protein, are also far from the RBS, and evolve the slowest. Intriguingly, the net charge of the negative branch has been increasing over time, whereas that of the positive and zero branches have been decreasing and constant, respectively. Importantly, multinomial logistic regression models for the dynamics of individual residue charge reveal that individual sites in the positive and negative branches are not each evolving toward an intermediate “zero” charge.

Why are there contrasting net charge patterns for each branch over time, in conjunction with differing rates of evolution? These results could imply the following: as the positive branch evolves more slowly than its negative counterpart, it is possible that the decrease in net charge over time seen in the positive branch is in response to an increase in net charge over time of the negative branch. Our results also indicate that the sites in the zero branch are highly conserved on average, that their net charge does not vary significantly over time, and that their site-wise fitted observed charge is generally zero.

In the context that the overall net charge of the HA for H1N1 viruses in humans has not increased over time [2], these results indicate that more localized charge may also be important in viral evolution. In fact, by considering those sites in the negative branch and thus closer to the RBS, the increase in net charge over time is likely due in part to the negative charge of the cell surface. An increase in net charge for this group that is nearest to the RBS could facilitate HA interactions with the negatively charged host cell surface. Furthermore, since the negative branch contains more sites that are evolving rapidly, this branch is probably immunodominant in comparison with the other two branches. Therefore, there is an apparent asymmetry between the positive and negative branches that is not revealed by functional selection experiments. This asymmetry could be possibly explained either by an intrinsic molecular property of the HA, or could result from host interactions. Indeed, it is thus possible that charge imposes constraints on these evolving viruses, or immune selection could impose such an asymmetry.

Perhaps the discrepancy between H1N1 and H3N2 viruses in the net charge patterns of HAs is simply a reflection of different constraints on HA biophysical factors such as stability, instead of a fundamental difference in the role of electrostatic charge in virion-host cell interactions between these two subtypes. In particular, it may be that the H1N1 HA is less tolerant to charge imbalances than the H3N2 HA, and therefore if a set of sites in the H1N1 HA is under pressure to become more positively charged, other sites must become more negatively charged in counterbalance. Deep mutational scans on H1N1 [22] and H3N2 [34] indicate that the H3N2 HA has a different mutational tolerance profile in comparison to the H1N1 HA. Further experiments should examine and quantify differences in tolerance to charge imbalance for H1N1 and H3N2 HAs to investigate the hypothesis of different tolerances to charge imbalance in these HAs. Furthermore, in natural sequences, H1N1 antigenic evolution is not as rapid as H3N2 antigenic evolution [35], and could indicate some differing constraints.

Another possible explanation for the observed differences in H1N1 and H3N2 HAs is that H3N2 and H1N1 virions have different NAs. Specifically, it is possible that having an increasingly positive HA could make it more difficult for NA to cleave new viruses from the cell, and it could be that the H3N2 NA protein is more tolerant of this than the H1N1 NA. To investigate this possibility and quantify the co-evolution of HA and NA charge, a combination of experiments and analyses of natural sequences are required.

However, it is possible that the patterns we see are due to other processes, or are simply covariates of other variables that we did not examine. Our inferences from the patterns of charge over time is limited, due to the fact that our study is retrospective and relies upon collected natural sequences of H1N1. The remarkable separation of residues into three branches, based on functional selection and expected charge, should be examined further. With H3N2 data, the separation into three branches is not as clear, and could be due to different relative mutational tolerances in the HA [34]. However, explorations of HA localized charge for this subtype are not as crucial, as the net charge of the whole H3N2 HA was found to increase over time [2]. Echoing Arinaminpathy and Grenfell [2], further experiments will be needed to elucidate the exact mechanisms involved in these processes, and to determine the role of electrostatic charge. Beyond H1N1 and H3N2 viruses, numerous subtypes of influenza circulate or have circulated among a variety of hosts. Analyses that examine the role of local charge could reveal unifying themes, with potential relevance for zoonosis and pandemic preparedness. Such analyses would require subtype-specific DMS data, in conjunction with temporally detailed natural sequences for specific hosts, and these analyses may become feasible with growing data availability.

Experiments to probe the mechanisms that give rise to patterns of charge could then guide informed decisions about vaccine design. The importance of these decisions, based on electrostatic interactions at the molecular level, would be especially relevant in the quest for broadly protective vaccines [15]. Preliminarily, our results might indicate that possible mechanisms for disease control could be to either limit the possibility of the negative branch increasing in charge, or, alternatively, limit the possibility of the positive branch decreasing in charge. Either of these options would require rational vaccine design, hopefully eliciting broadly cross-reactive antibodies.

Furthermore, understanding how functional selection and expected charge co-vary for different pathogens’ proteins under immune selection could help characterize, quantify, and generalize the role of charge in evolutionary dynamics of pathogens. Quantifying these properties for other proteins is also important, and we showed that beta-lactamase protein gives rise to different patterns than the H1N1 HA. Given this stark contrast, it is possible that the pattern seen for the H1N1 HA is the result of evolution due to immune selection. If this is the case, signatures of evolutionary pressures on other proteins could be detected by functional selection and charge. Thus, independent of the specifics of a particular protein, a “null” distribution of sites according to functional selection and expected charge would be quite valuable in principle. However, such a distribution would be very difficult to obtain. This would require understanding which random sequences of amino acids can properly fold, and with these, perform DMS experiments to quantify amino acid preferences at each site.

It is striking that simple measures of functional selection and expected charge are able to discriminate sites that have higher evolutionary rates, particularly when these measures are independent of antibody interactions and are based solely upon cell entry and viral replication. That these sites then show an increase in net charge over time is even more intriguing. Overall, our results possibly implicate localized charge on the HA protein in evolutionary dynamics, particularly in HA-cell host interactions. We could speculate that certain regions on the HA protein could be under pressure to gain charge in order to facilitate attachment to the cell surface, whereas others would be under pressure to decrease in charge in response to such an increase.

## Materials and methods

### Deep mutational scan (DMS) data

The DMS generates amino acid preferences for each site, which are normalized enrichment. A preference of 0 denotes complete depletion of that particular amino acid and a preference of 1 denotes a complete depletion of all other amino acids in favor of that particular amino acid. For H1N1 HA data using dmstools2 [29], these preferences can be calculated (see example of Doud and Bloom [22] in dmstools2 documentation) for the HA using a Bayesian approach, in order to account for the possibility of errors in sequencing. We obtained the HA amino acid preferences from the Bloom lab GitHub repository.

To quantify purifying selection due to function (“functional selection”), we use a transformation of Shannon’s evenness, a statistic often used in ecological systems to quantify diversity [36]. Denoting *p*_*i*,*a*_ as the preference for amino acid *a* ∈ *A* at site *i*, we defined the functional selection coefficient as

$$\begin{array}{c} S_{i}=1-\sqrt{-\frac{1}{\ln20}\sum_{a\in A}p_{i,a}\ln p_{i,a}}. \end{array}$$ (1)

*S*_*i*_ is simply a transformation of Shannon’s evenness, so that *S*_*i*_ ≈ 0 if there are few functional constraints, and *S*_*i*_ ≈ 1 if there are strong functional constraints.

In addition, by using the same DMS data, we also computed the expected charge at each site, essentially giving the expected charge *C*^(*i*)^ at each site *i* due to functional constraints on the HA protein. That is,

$$\begin{array}{c} C^{\left(i\right)}=\sum_{a\in A}p_{i,a}c_{a}, \end{array}$$ (2)

where *c*_*a*_ ∈ {−1, 0, 1} is the charge of the amino acid *a* ∈ *A*. Here, we assumed that Arginine, Histidine, Lysine, Aspartic Acid, and Glutamic Acid are the only charged amino acids with a charge magnitude of 1, and with the first three positive and the last two negative, just as was done by Arinaminpathy and Grenfell [2]. Note that for beta-lactamase analyses, we used the amino acid preferences as given in Bloom [37].

The three branches shown in Fig 2A were separated by visual inspection, with the negative branch consisting of sites *i* where *C*^(*i*)^ < 0.04 and *S*_*i*_ < 0.05 − 0.7*C*^(*i*)^, the positive branch consisting of sites *i* where *C*^(*i*)^ > 0.041 and *S*_*i*_ < 0.02 + 0.5*C*^(*i*)^, and the zero branch containing sites that are in neither other branch.

### Relative solvent accessibilities (RSAs) and distances to receptor binding site (RBS)

For HA residues, we computed relative solvent accessibilities (RSAs) using dmstools2 [29], and distances from the centroid of each residue to the centroid of the receptor binding site (RBS) were obtained from PyMOL 2.2.0 [23] using the HA monomer. The pdb file for the HA monomer was generated with pdb-tools [38] by selecting chain A of the trimer. Note that RSAs and distances to RBS could not be computed for certain sites in the reference DMS sequence, as these values rely upon the closest available 3-dimensional H1N1 HA protein (used in Doud and Bloom [22]). Values for RSAs and distances to the RBS are also missing since we consider HA1 to contain the signal peptide, and some HA2 sites are not identified on the 3-dimensional H1N1 HA protein. To determine which sites on the HA are in the receptor binding site (RBS), we followed [24] (in H3 numbering) and comprised the RBS of the 130-loop (residues 135 to 138), the 190-helix (residues 190 to 198), the 220-loop (residues 221 to 228), and the RBS base (residues 98, 153, 183, and 195). To convert from H3 numbering to sequential numbering, we followed the conversion scheme given in Doud and Bloom [22]. To compute RSAs for beta-lactamase, we followed Bloom [37] and also used dmstools2 [29].

### Glycosylation

Under no immune selection, the probability of glycosylation at site *i* was calculated by finding the probability of having the Asn−*X*−Ser/Thr sequon [39] (where *X* is not proline) assuming site independence. This assumption is necessary and implicit as DMS data only consider single residue changes on a specific background HA protein. In particular, these data do not consider epistatic interactions between residues, including any such interactions that would enrich Asn−*X*−Ser/Thr sequons. Therefore, the estimate of the glycosylation probability at site *i* is

$$\begin{array}{c} P\left\{{\text{Glycosylation}}_{i}\right\}=P\left\{{\text{Asn}}_{i}\right\}\left(1-P\left\{{\text{Pro}}_{i+1}\right\}\right)\left(P\left\{{\text{Ser}}_{i+2}\right\}+P\left\{{\text{Thr}}_{i+2}\right\}\right). \end{array}$$

### Analyses with natural sequences

For our sequence analyses with H1N1 between 1918 to 2008, we obtained all available 1744 H1 sequences in human hosts from http://www.fludb.org, of the Influenza Research Database (IRD) [28], and aligned these using MUSCLE in fludb. For net charge computations, we again followed Arinaminpathy and Grenfell [2]. That is, we summed the individual charges of amino acids across the alignments, where Arginine, Histidine, and Lysine have a charge of 1, Aspartic acid and Glutamic acid have a charge of −1, and all other amino acids have a charge of 0. Given ambiguous amino acids (*i.e.*, X, B, Z) in a particular sequence, we assumed that the indicated respective choices of residues at that site were uniformly distributed and computed the expected charge at that site for that sequence. These temporal trends, along with other graphs, were visualized with the ggplot2 package [40] of R [31].

### Evolutionary rates

To compute evolutionary rates of amino acid substitutions, we selected the 958 unique sequences between 1918 and 2008, aligned these within fludb, and used the Rate4Site algorithm [27]. To statistically test differences in distributions of evolutionary rates and other HA characteristics across groups of residues, we used the non-parametric Kolmogorov-Smirnov test with ks.test from R [31] (note that the p-values from these tests are sometimes not exact, due to ties). For beta-lactamase, we used the sequences given in Bloom [37] and computed rates using the Rate4Site algorithm [27].

### Fitted observed per-residue charge

Across 1918 to 2008, the charge in a given year, *i.e.*, “yearly charge”, may vary for individual sites. In order to infer per-residue yearly charge from natural sequence data in the multiple sequence alignment, we fit multinomial statistical models. To compute the site-wise fitted observed charge from natural sequences, we used maximum likelihood estimation to obtain fitted observed charge over time at each site. If more than one charge value was present in the set of sequences at a particular site, we used the nnet package [30] in R [31] to fit a multinomial logistic regression model at each of the 565 sites, in order to obtain the probability *f*_*i*,*c*_(*t*) of having the charge *c* ∈ {−1, 0, 1} at site *i* at time *t*. Otherwise, we set *f*_*i*,*c*_(*t*) = 1 and *f*_*i*,*c*_(*t*) = 0, respectively, for the values of *c* that occurred in all sequences and for the values of *c* that occurred in none, respectively. Note that if there was ambiguity in a given sequence about its residue at a certain position, that residue was omitted when fitting multinomial models, in contrast to net charge computations. Then, we calculated the fitted observed charge $C_{P}^{\left(i\right)}\left(t\right)$ at site *i* at time *t* as

$$\begin{array}{c} C_{P}^{\left(i\right)}\left(t\right)=\sum_{c\in \{-1,0,1\}}cf_{i,c}\left(t\right). \end{array}$$ (3)

## Supporting information

S1 Appendix Supplementary analyses and results. (PDF)

S1 Fig Yearly means of net charge over time in negative (*A*), positive (*B*), and zero (*C*) branches. This figure is just as in Fig 2A of the main text, but employing yearly means instead of all sequences. Here, yearly means are averages of the charge of all sequences in a given year, giving a single charge value for each year that at least one sequence is available. This approach minimizes any potential biases introduced due to uneven sampling in different years.
(PDF)

S2 Fig Net charge over time in (*A*) negative (*P* < 2.2 × 10^−16^), (*B*) positive (*P* < 2.2 × 10^−16^), and (*C*) zero (*P* < 2.2 × 10^−16^) branches of HA1 subunit. This figure is just as in 1A, except only the immunodominant HA1 subunit is being considered.
(PDF)

S3 Fig Yearly means of net charge over time in negative (*A*), positive (*B*), and zero (*C*) branches of HA1 subunit. This figure compliments S2 Fig and is analogous to S1 Fig except that only residues in the HA1 subunit are being considered in contrast to the whole HA in S1 Fig.
(PDF)

S4 Fig Net charge of H1N1 HA over time. (*A*) Net charge from 1918 to 2008 in 1741 H1N1 sequences from human hosts, obtained from the IRD [28] at http://www.fludb.org. This figure confirms that the net charge of the HA H1N1 has not varied significantly since its introduction in 1918. (*B*) as in (A), but with yearly means instead.
(PDF)

S5 Fig Relative Solvent Accessibilities (RSAs) across branches and HA domains. RSAs for (*A*) HA1 and (*B*) HA2, with (*C*) empirical CDFs for each branch in each domain.
(PDF)

S6 Fig Beta-lactamase functional selection as a function of expected charge, overlaid with both RSA and evolutionary rates. (*A*) RSA values for beta-lactamase residues overlaid on the functional selection-expected value plot for these residues. (*B*) Normalized conservation scores, calculated with the Rate4Site algorithm [27], overlaid on the plot of selection as a function of expected charge, for beta-lactamase.
(PDF)

S7 Fig Probabilities of glycosylation per site, with non-zero probabilities plotted on log-scale to increase contrast in values. These values are plotted on the functional selection-expected value plot for HA residues on (*A*) a linear scale, and on (*B*) a log-scale. The probabilities of glycosylation were computed using DMS data and assuming independence of sites (see Materials and methods).
(PDF)

S8 Fig Sites shown to influence binding, according to Hensley and coauthors [1], overlaid on the functional selection-expected value RSA plot for HA residues in HA1. The sites that change binding are denoted with large circles, whereas those other residues that were not so identified are depicted as points. Single mutations are these sites led to mutants denoted as “better binders” or “worse binders” by Hensley [1], Table S2. In H3 numbering, the sites affecting binding are 128, 129, 164, 165, 166, 158, 156, 192, 193, 196, 198, 143, 224, 244, 74, 75, 119, 162, 93, 145 [1].
(PDF)

S9 Fig Alternative clustering scheme, using hierarchical clustering and Ward’s method with Euclidean distance (instead of distance squared) on unscaled data. The three colours denote the three clusters identified through this alternative scheme.
(PDF)

S10 Fig Temporal charge trends for the clusters determined by the alternative clustering method, with clusters shown in S9 Fig. The *red*, *blue*, and *green* colours depict the positive, negative, and zero branches identified through hierarchical clustering with Ward’s method in S8 Fig. (*A*)–(*B*) Temporal trends by clusters with all sequences for (*A*) the whole HA and (*B*) HA1. (*C*)–(*D*) Yearly means for each cluster, for (*A*) the whole HA and (*B*) HA1.
(PDF)

S11 Fig Empirical cumulative distributions for each branch and each HA domain with the alternative clustering scheme. (*A*)–(*C*) are for distributions of evolutionary rates for (*A*) the whole HA, (*B*) HA1, and (*C*) HA2. (*D*)–(*F*) are for distributions of distances to the RBS for (*D*) the whole HA, (*E*) HA1, and (*F*) HA2. (*G*)–(*I*) are for distributions of RSAs for (*G*) the whole HA, (*H*) HA1, and (*I*) HA2. For the whole HA and RBS distributions, the positive-negative and negative-zero branches are significantly different (*D* = 0.2207, *P* = 0.003768 and *D* = 0.26109, *P* = 9.992 × 10^−5^, respectively), whereas the positive and zero branches are not significantly different (*D* = 0.10194, *P* = 0.2699). For RSA distributions, the positive-negative, positive-zero, and negative-zero branches are all significantly different (*D* = 0.17977, *P* = 0.03591; *D* = 0.57619, *P* < 2.2 × 10^−16^; and *D* = 0.62497, *P* < 2.2 × 10^−16^, respectively). For evolutionary rates, all the differences in branches are also significant (*D* = 0.17297, *P* = 0.02768 [positive-negative]; *D* = 0.32241, *P* = 2.215 × 10^−10^ [positive-zero]; and *D* = 0.38207, *P* = 1.837 × 10^−10^ [negative-zero]). The statistical significance of differences is similar for comparisons of branches with only HA1 sites, at a significance level of *α* = 0.05.
(PDF)

S1 Analyses This file contains files and codes for our analyses. (ZIP)
