# Supplementary material for: Implications of localized charge for human influenza A H1N1 hemagglutinin evolution: Insights from deep mutational scans
Source: PLoS Comput Biol. 2020 Jun 25;16(6):e1007892. doi: 10.1371/journal.pcbi.1007892 (PMC7316228; doi:10.1371/journal.pcbi.1007892)
Supplement: S1 Appendix — (PDF) [file pcbi.1007892.s001.pdf]

Supplementary Information: Implications of localized charge for human influenza A H1N1 hemagglutinin evolution: Insights from deep mutational scans

Chadi M. Saad-Roy*† Nimalan Arinaminpathy‡ Ned S. Wingreen†§
Simon A. Levin¶ Joshua M. Akey† Bryan T. Grenfell*¶‖**

Supplementary Analyses and Results

Performing the same suite of analyses on the clusters using hierarchical clustering reveals that the major results on temporal trends of charge remain (S10 Fig). Indeed, this "new" negative branch exhibits a strong increase in charge, whereas the "new" positive branch displays a decrease in charge (S10A Fig), and the "new" zero branch also exhibits a decrease in charge (S10A Fig). Furthermore, these same patterns are also seen when we consider just the HA1 subunit (S10B Fig). Lastly, these results are similar if yearly means are considered instead of all sequences (S10C, D Fig). The smaller decrease in charge of the positive branch with this alternative clustering is likely due to different classifications between the positive and zero branch with this new clustering.

When the empirical CDFs of the corresponding distributions are examined (S11 Fig), it is apparent that our new analyses give largely similar results for the whole HA and for the HA1 subunit. It is clear that the negative branch evolves the fastest, followed by the positive branch, and then the zero branch (S11A, B Fig). With respect to distances to the RBS, the negative branch again seems closer to the RBS, when sites in the whole HA are considered (S11D Fig) or only sites in the HA1 subunit are considered (S11E Fig). Moreover, the positive and negative branches are substantially more solvent exposed than the zero branch (S11G, H Fig), with the negative branch seeming slightly more solvent exposed than the positive branch. Another interesting observation that arises from this alternative clustering is the comparison of evolutionary rates on branches of the HA1 and HA2 subunits (S11B, C Fig). It seems that most of the differences in evolutionary rates are driven by residues in the HA1 subunit. Given

*Corresponding authors: csaadroy@princeton.edu (CMSR), grenfell@princeton.edu (BTG)
†Lewis-Sigler Institute for Integrative Genomics, Princeton University, Princeton, NJ, USA
‡MRC Centre for Global Infectious Disease Analysis, Department of Infectious Disease Epidemiology, School of Public Health, Imperial College London, London, UK
§Department of Molecular Biology, Princeton University, Princeton, NJ, USA
¶Department of Ecology and Evolutionary Biology, Princeton University, Princeton, NJ, USA
‖Woodrow Wilson School of Public and International Affairs, Princeton University, Princeton, NJ, USA
**Division of International Epidemiology and Population Studies, Fogarty International Center, National Institutes of Health, Bethesda, MD, USA

that HA1 is immunodominant in contrast to HA2, this further supports the hypothesis that immune selection is driving an increase in charge on the negative branch. Overall, the trends are relatively conserved with this alternative clustering.
