# Supplementary figures and images for: Implications of localized charge for human influenza A H1N1 hemagglutinin evolution: Insights from deep mutational scans
Source: PLoS Comput Biol. 2020 Jun 25;16(6):e1007892. doi: 10.1371/journal.pcbi.1007892 (PMC7316228; doi:10.1371/journal.pcbi.1007892)

**A**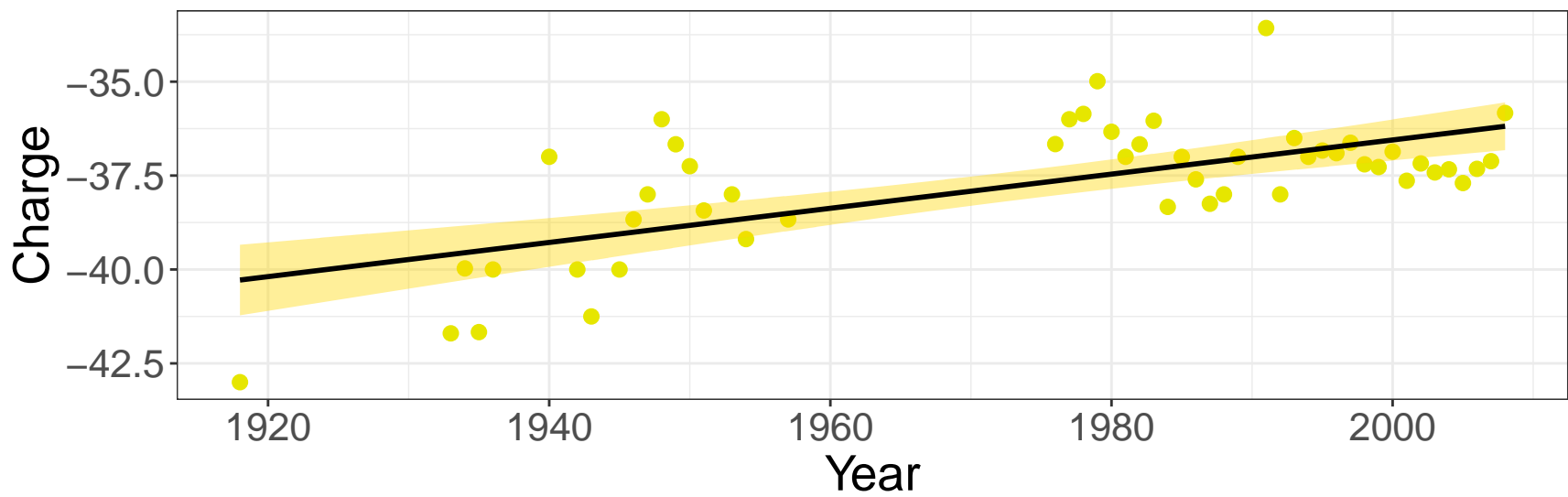**B**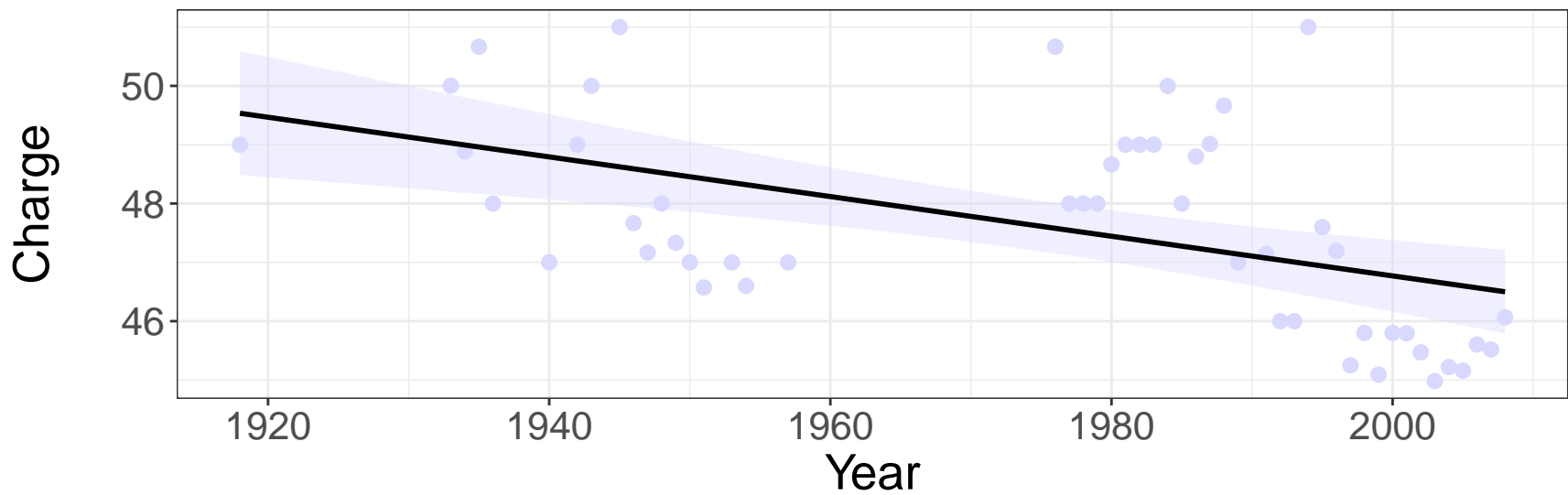**C**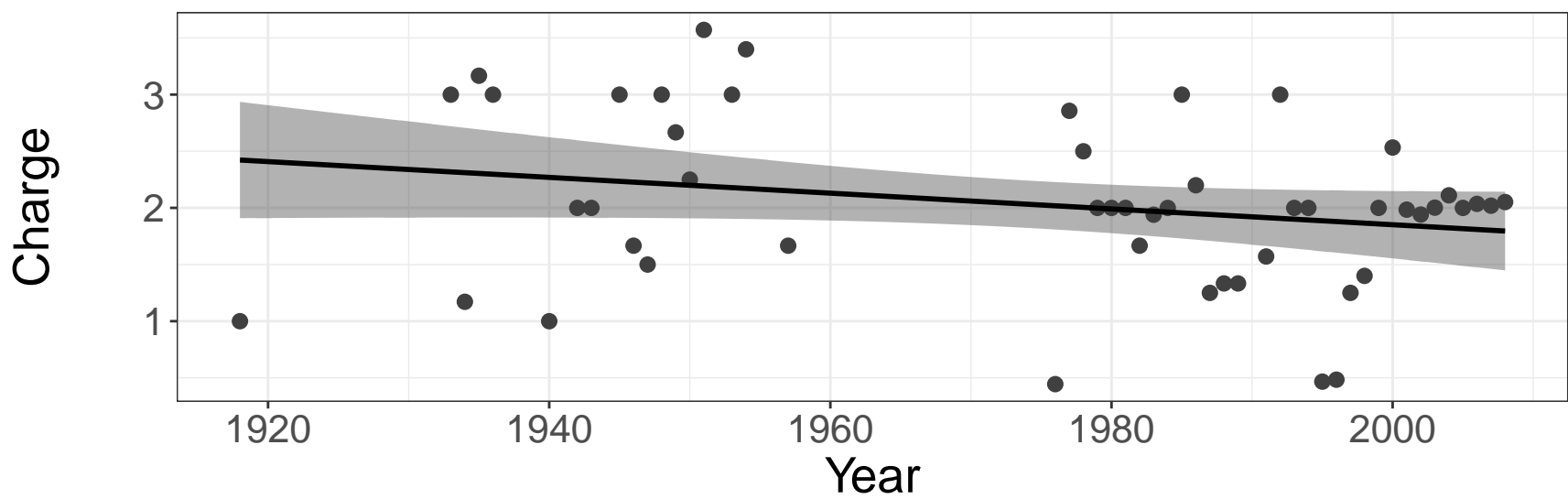

Supplement: S1 Fig — This figure is just as in Fig 2A of the main text, but employing yearly means instead of all sequences. Here, yearly means are averages of the charge of all sequences in a given year, giving a single charge value for each year that at least one sequence is available. This approach minimizes any potential biases introduced due to uneven sampling in different years. (PDF) [file pcbi.1007892.s002.pdf]

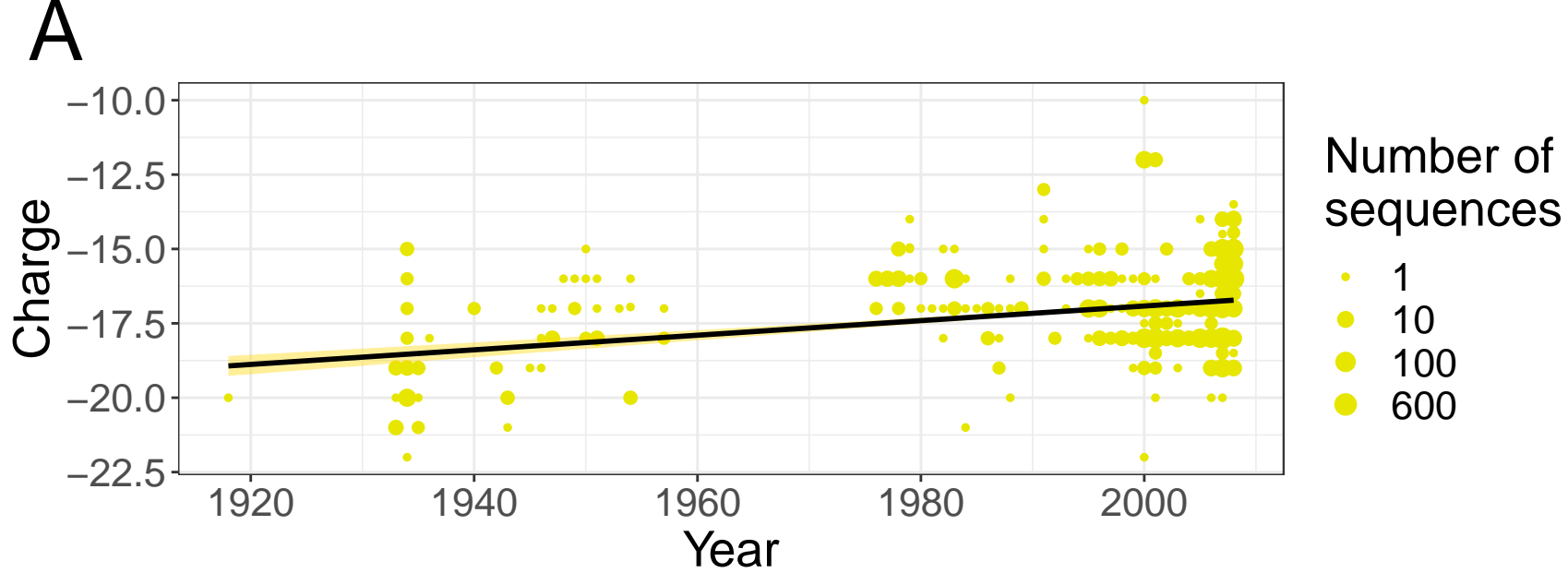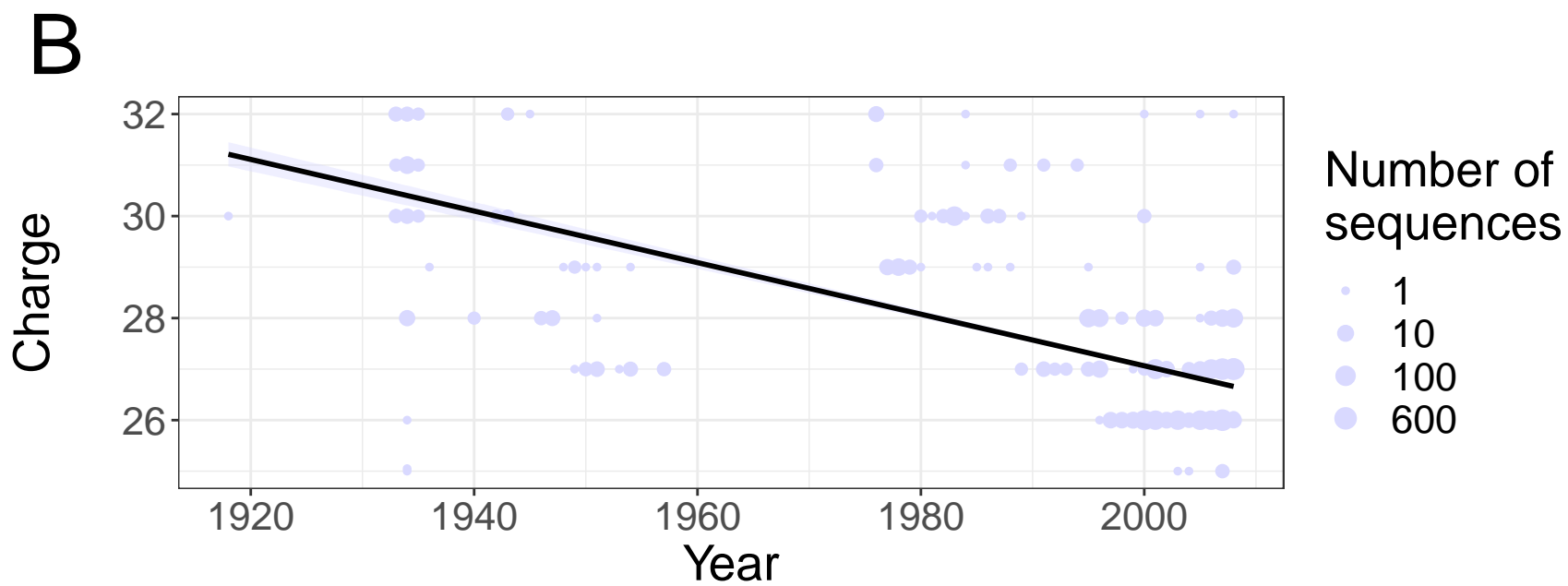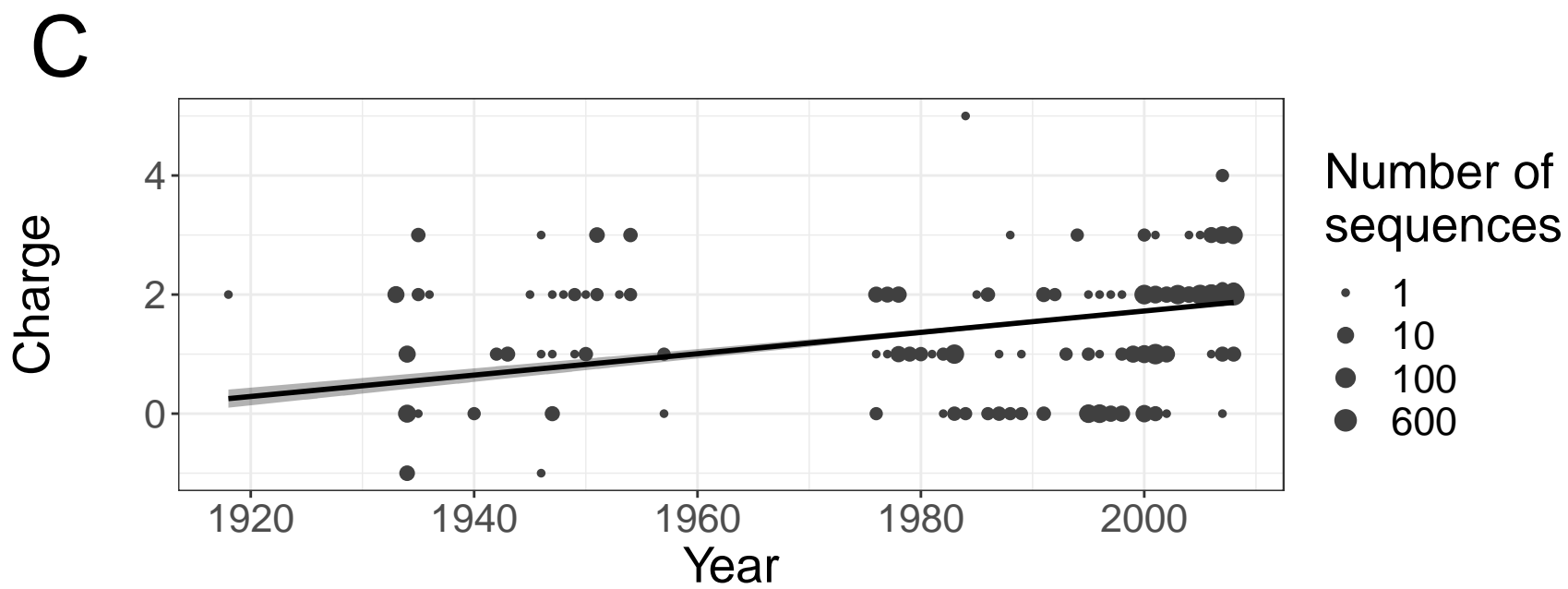

Supplement: S2 Fig — This figure is just as in 1A, except only the immunodominant HA1 subunit is being considered. (PDF) [file pcbi.1007892.s003.pdf]

**A**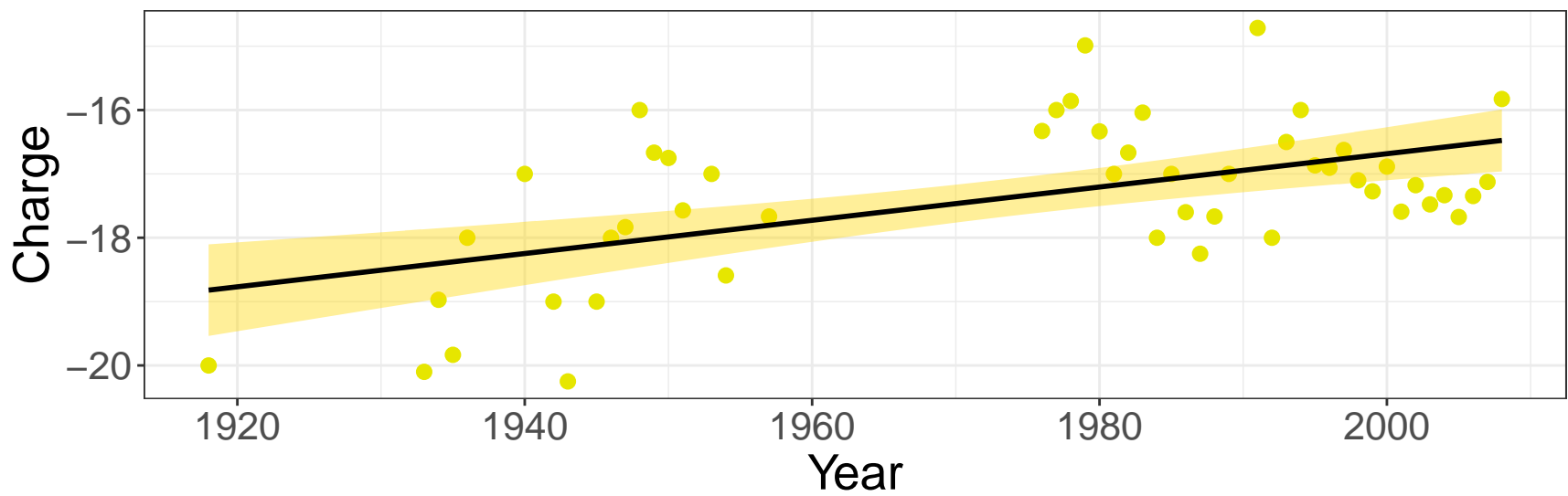**B**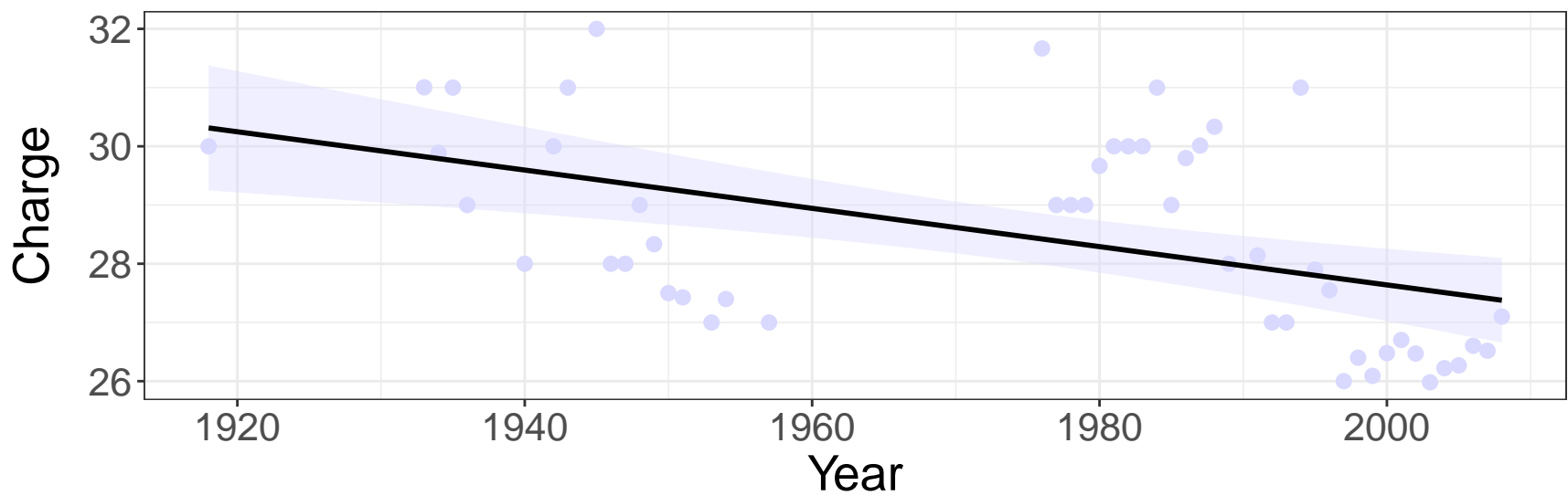**C**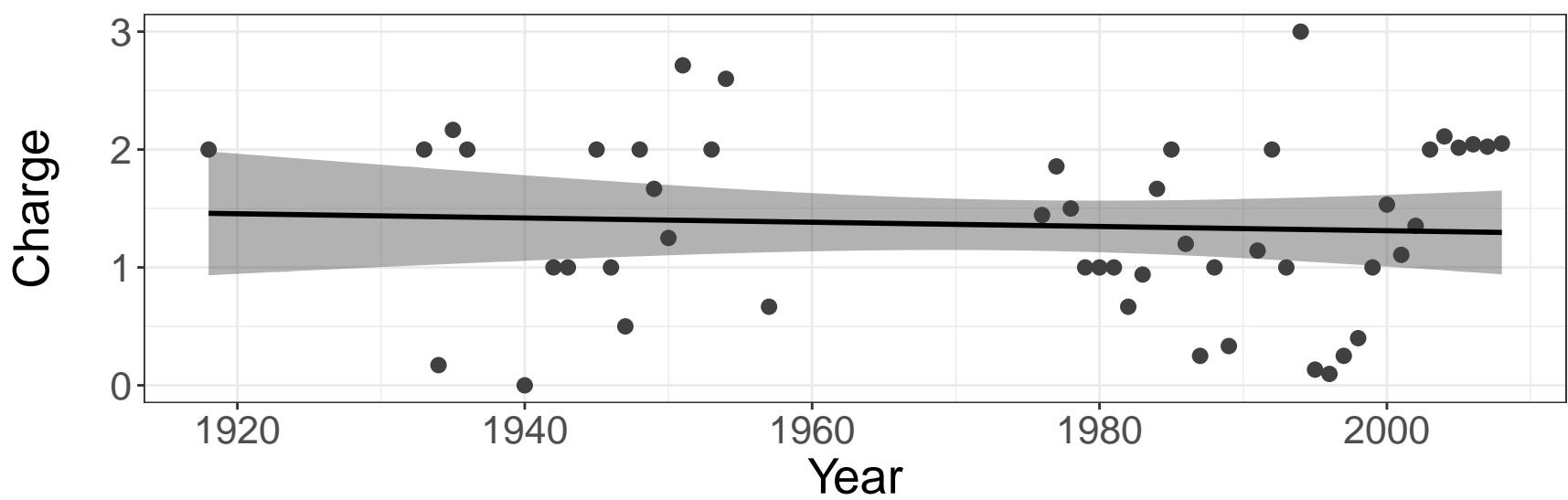

Supplement: S3 Fig — This figure compliments S2 Fig and is analogous to S1 Fig except that only residues in the HA1 subunit are being considered in contrast to the whole HA in S1 Fig. (PDF) [file pcbi.1007892.s004.pdf]

**A**

Charge

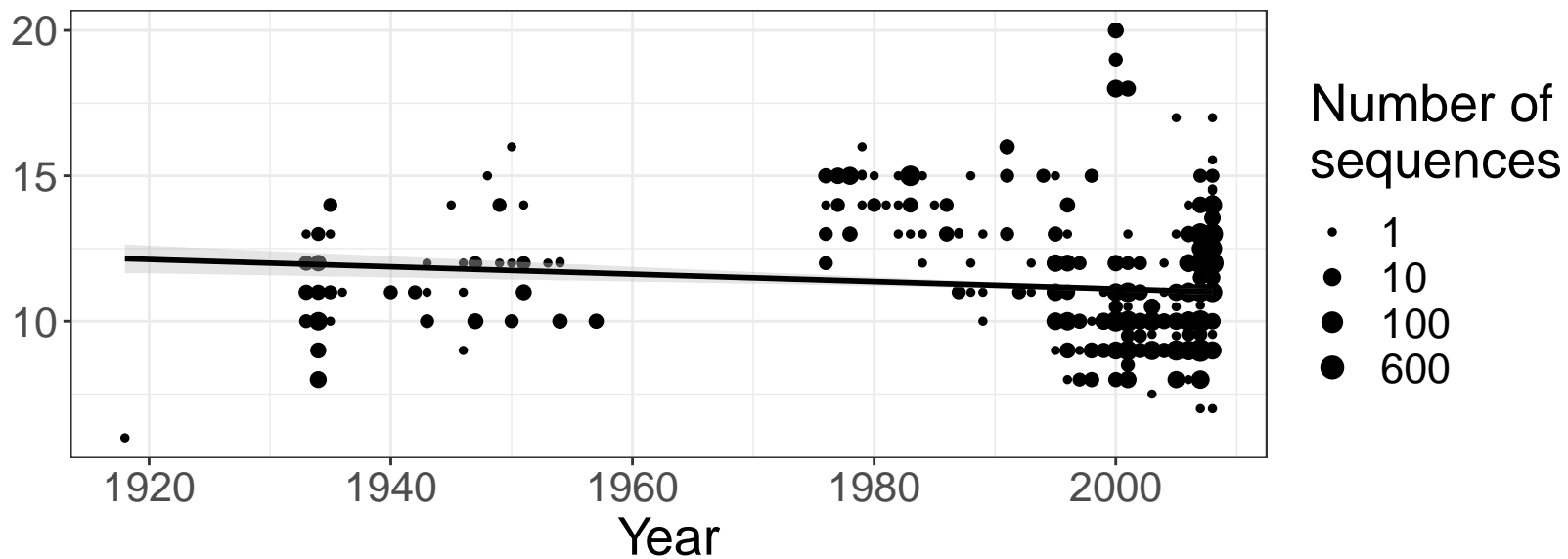**B**

Charge

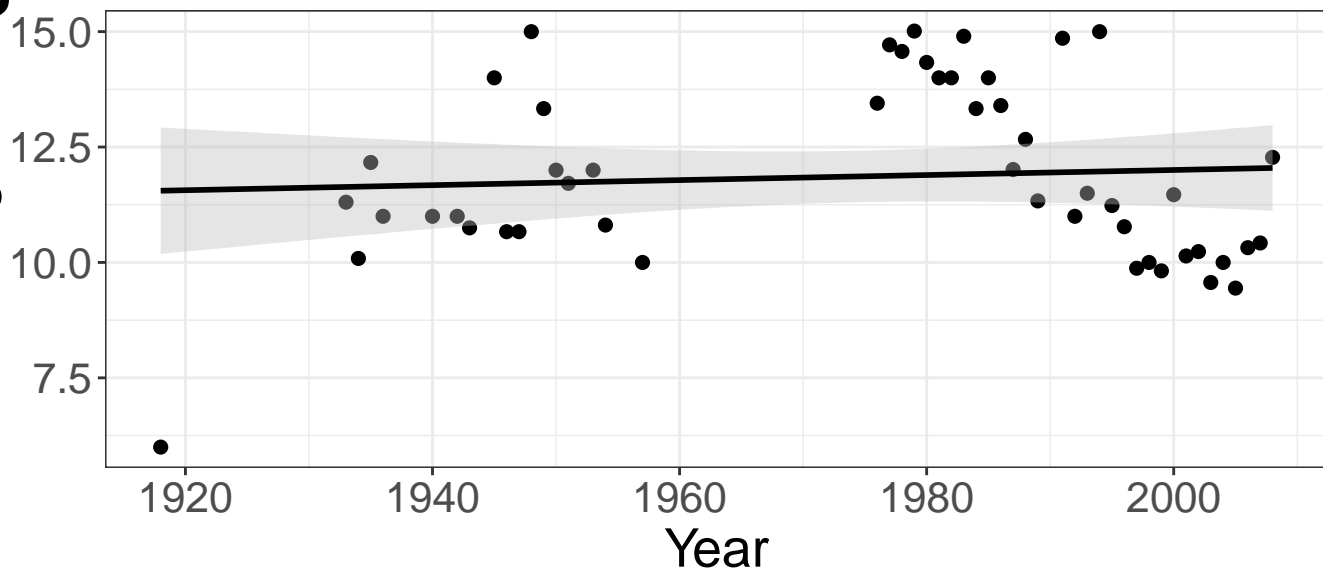

Supplement: S4 Fig — (A) Net charge from 1918 to 2008 in 1741 H1N1 sequences from human hosts, obtained from the IRD [28] at http://www.fludb.org. This figure confirms that the net charge of the HA H1N1 has not varied significantly since its introduction in 1918. (B) as in (A), but with yearly means instead. (PDF) [file pcbi.1007892.s005.pdf]

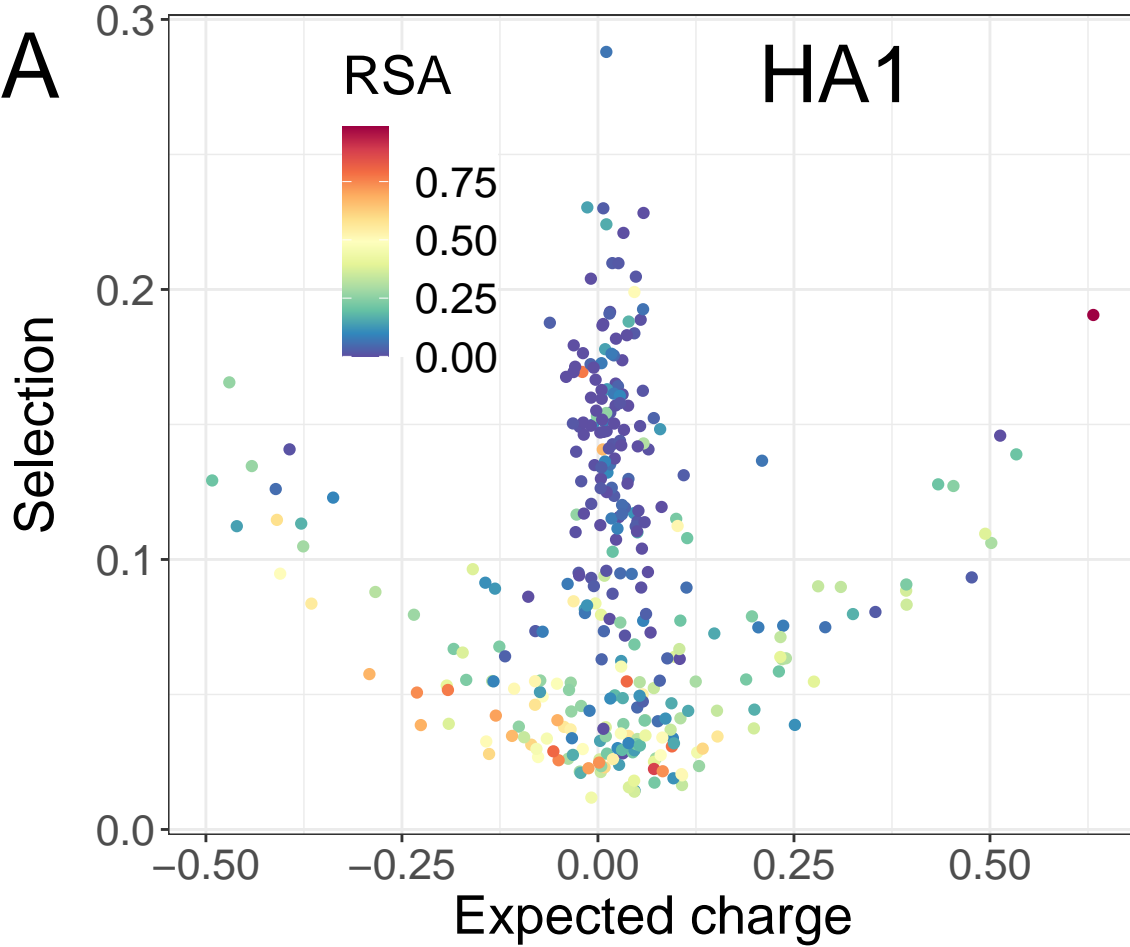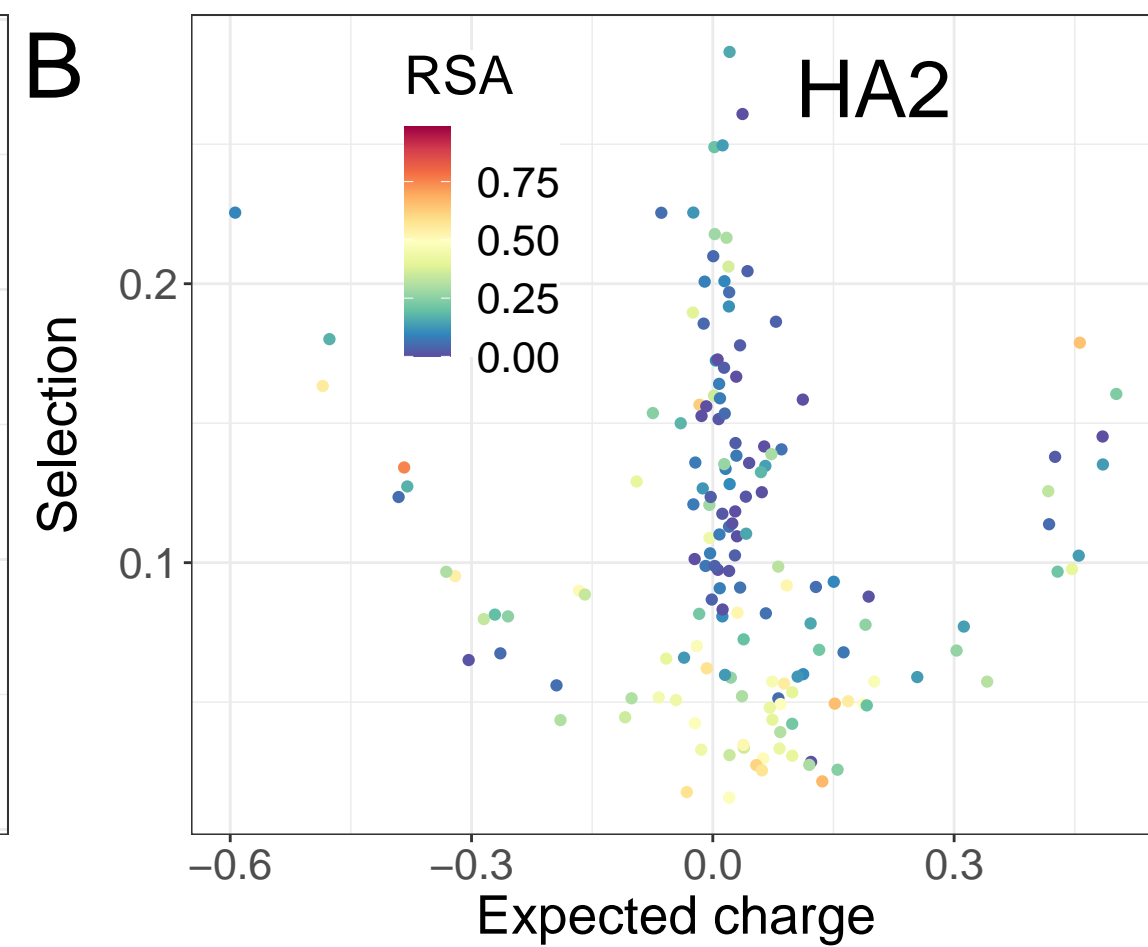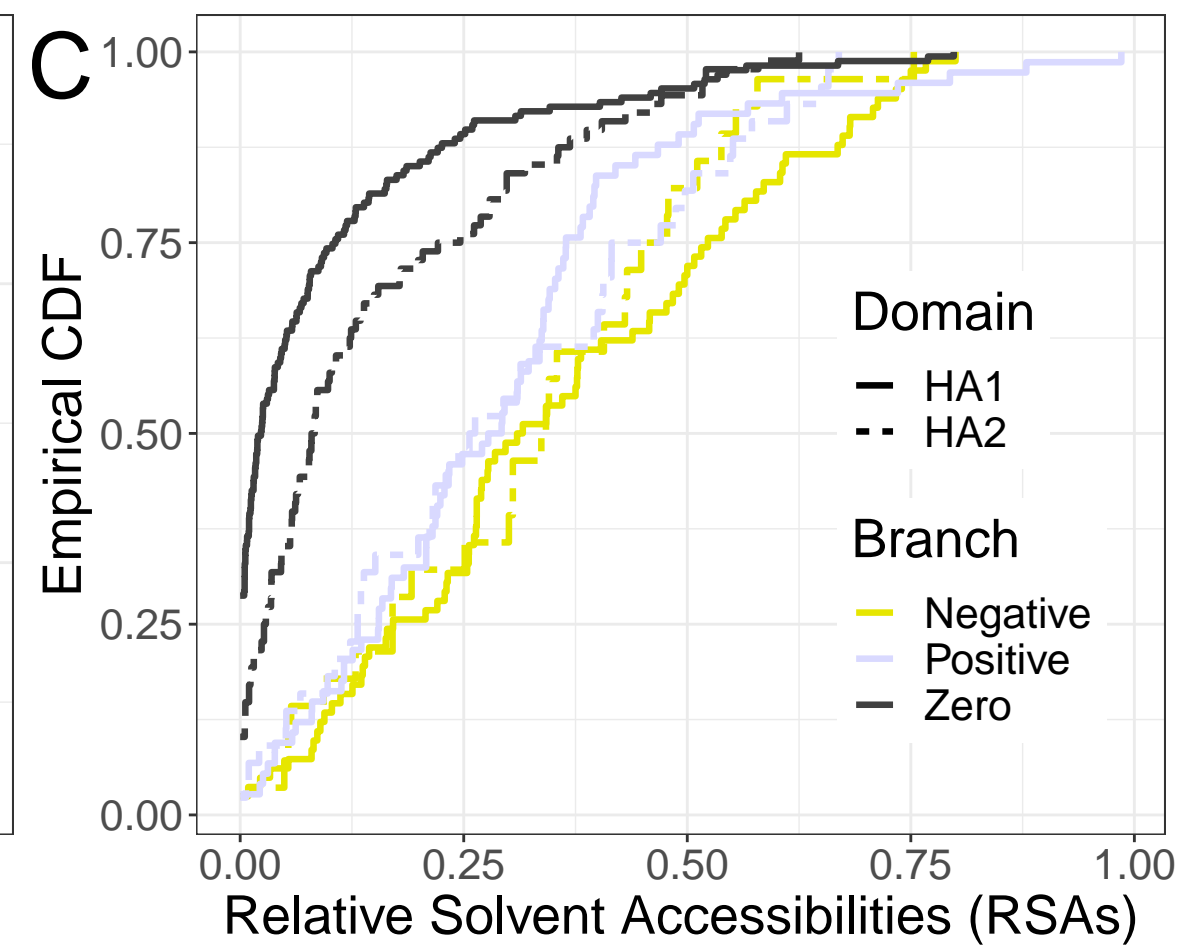

Supplement: S5 Fig — RSAs for (A) HA1 and (B) HA2, with (C) empirical CDFs for each branch in each domain. (PDF) [file pcbi.1007892.s006.pdf]

**A**

Functional selection

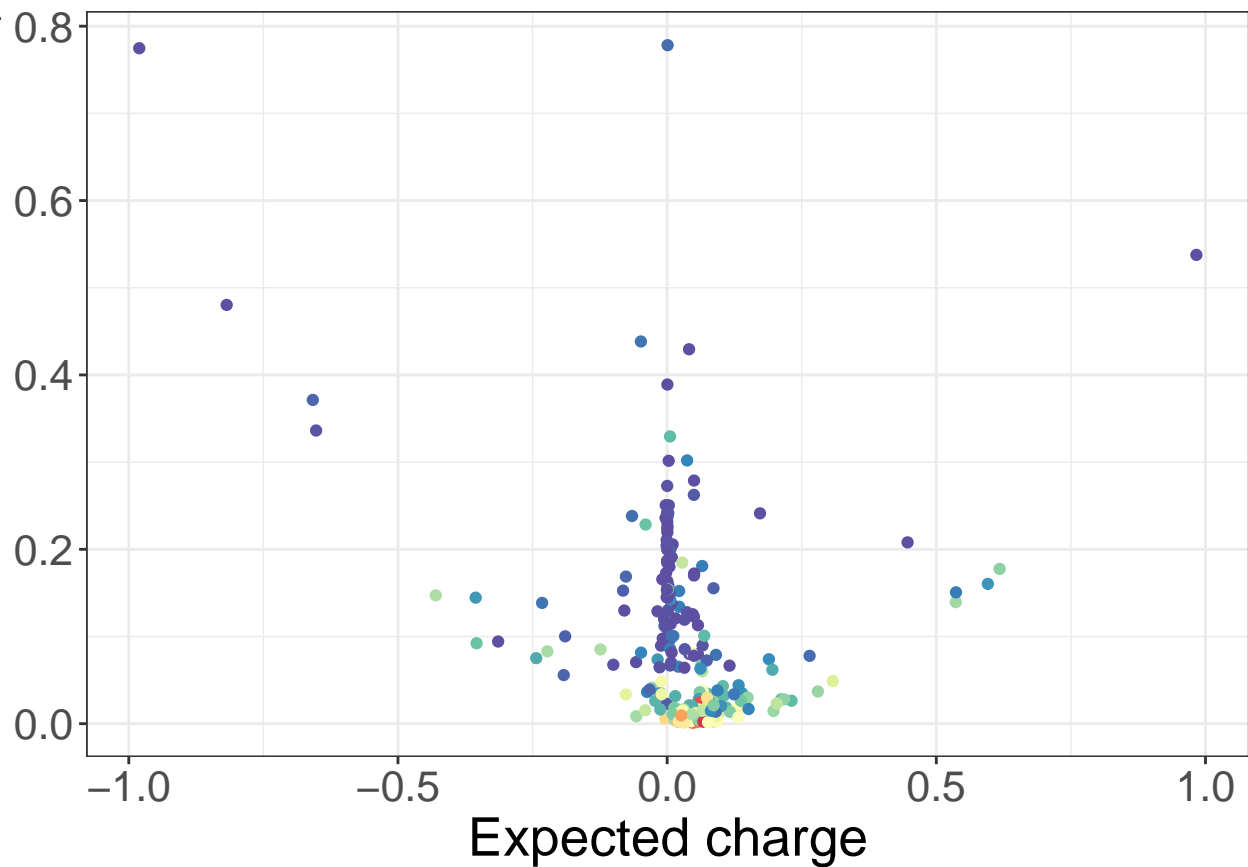**B**

Functional selection

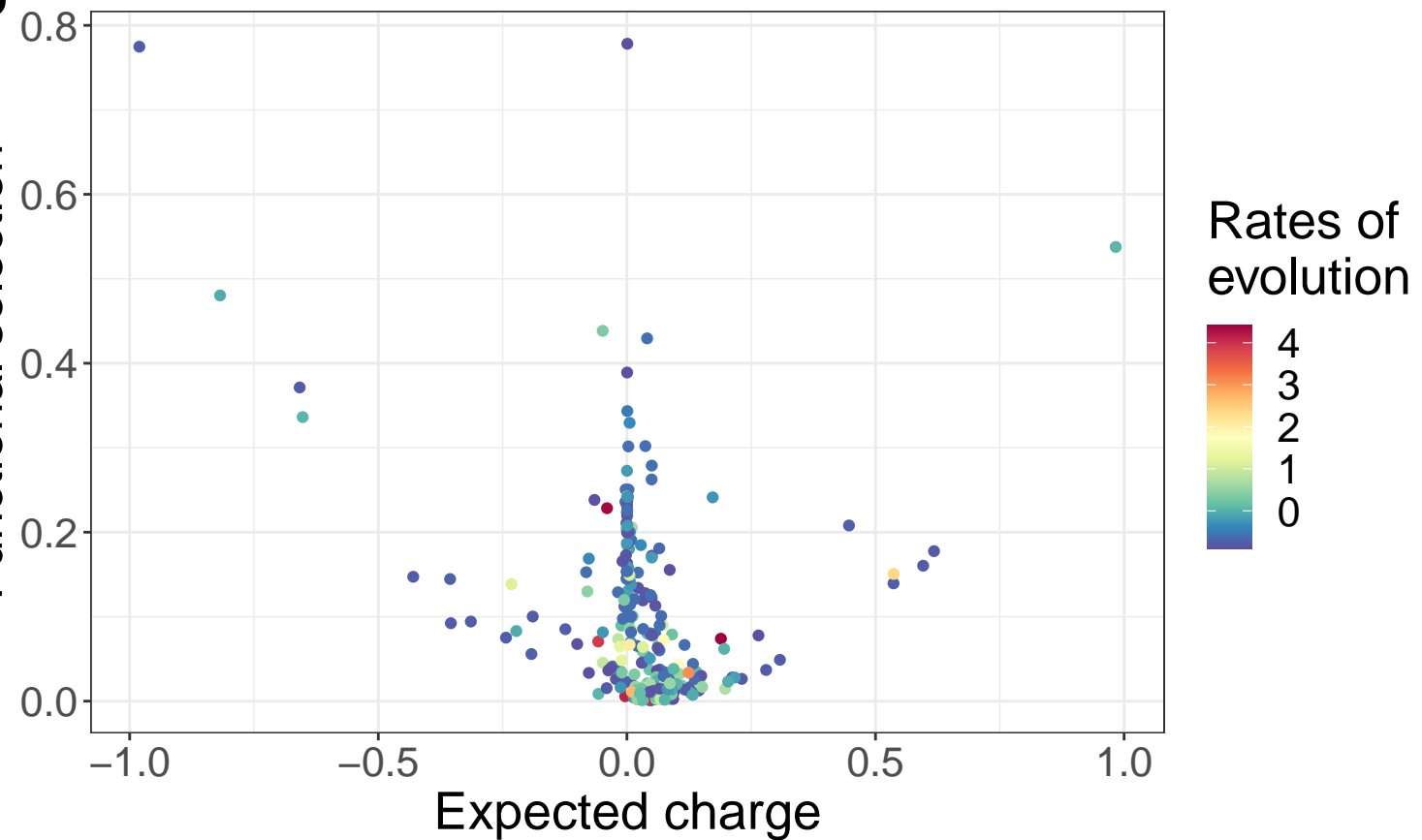

Supplement: S6 Fig — (A) RSA values for beta-lactamase residues overlaid on the functional selection-expected value plot for these residues. (B) Normalized conservation scores, calculated with the Rate4Site algorithm [27], overlaid on the plot of selection as a function of expected charge, for beta-lactamase. (PDF) [file pcbi.1007892.s007.pdf]

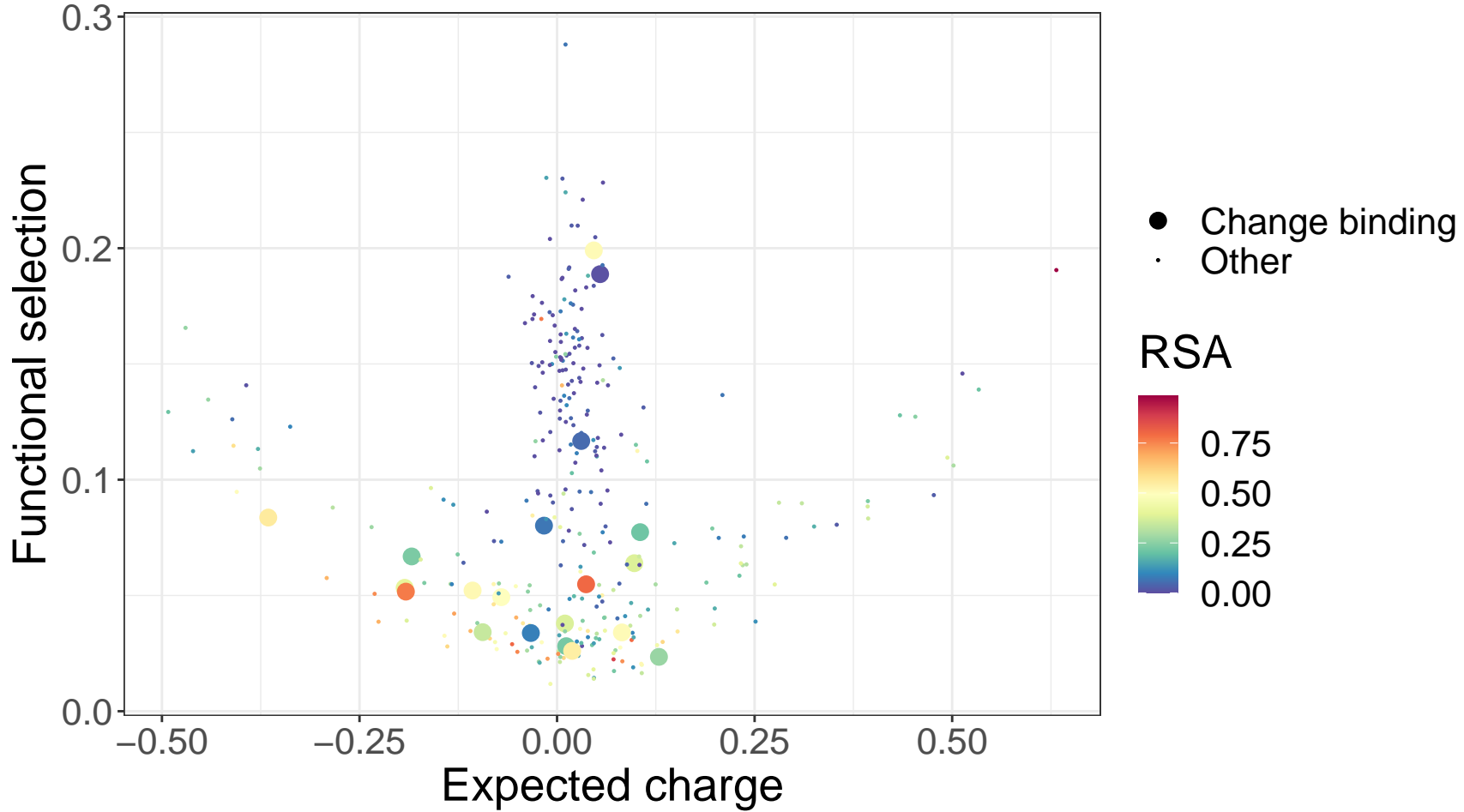

Supplement: S8 Fig — The sites that change binding are denoted with large circles, whereas those other residues that were not so identified are depicted as points. Single mutations are these sites led to mutants denoted as “better binders” or “worse binders” by Hensley [1], Table S2. In H3 numbering, the sites affecting binding are 128, 129, 164, 165, 166, 158, 156, 192, 193, 196, 198, 143, 224, 244, 74, 75, 119, 162, 93, 145 [1]. (PDF) [file pcbi.1007892.s009.pdf]

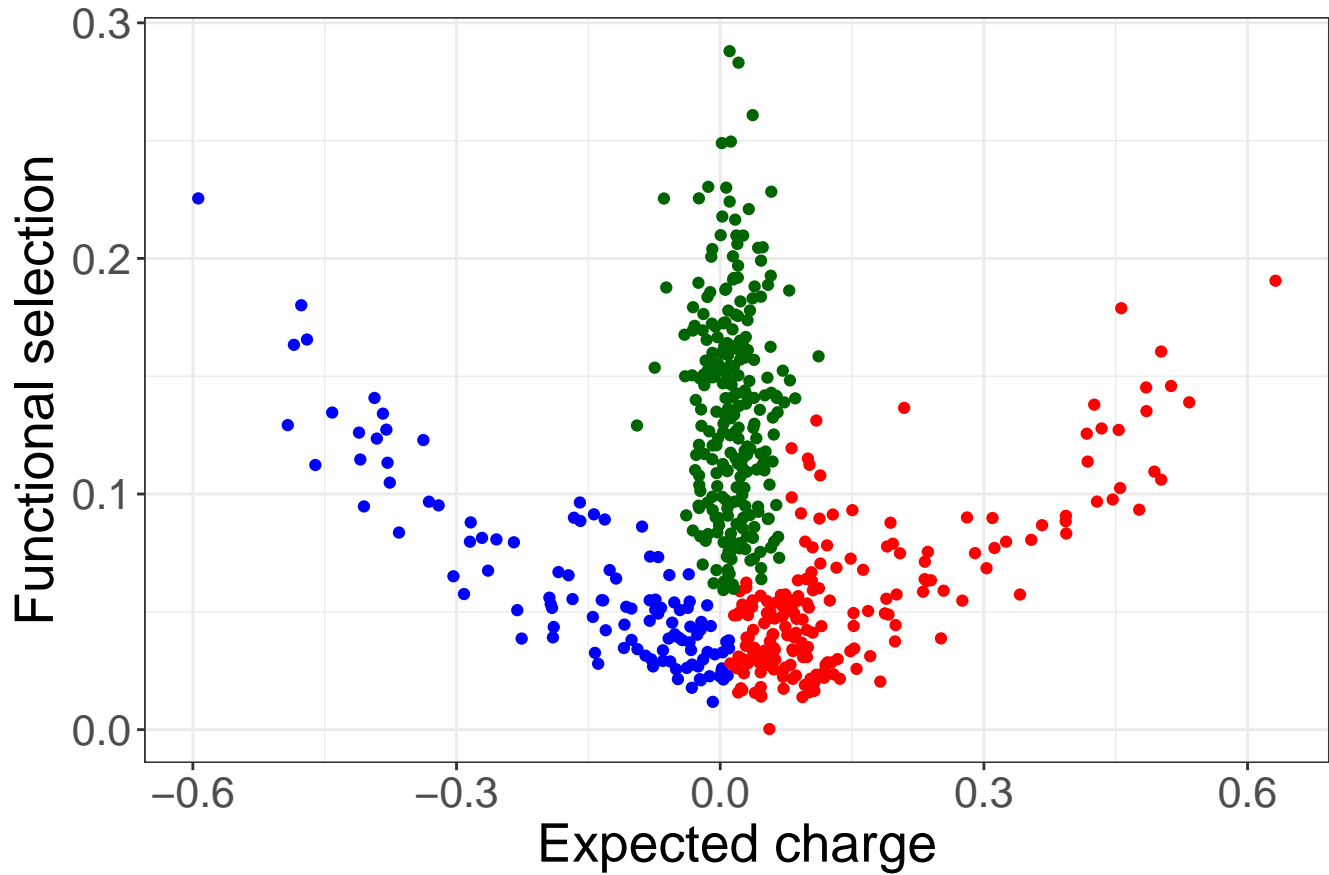

Supplement: S9 Fig — The three colours denote the three clusters identified through this alternative scheme. (PDF) [file pcbi.1007892.s010.pdf]

**A** All sequences, whole HA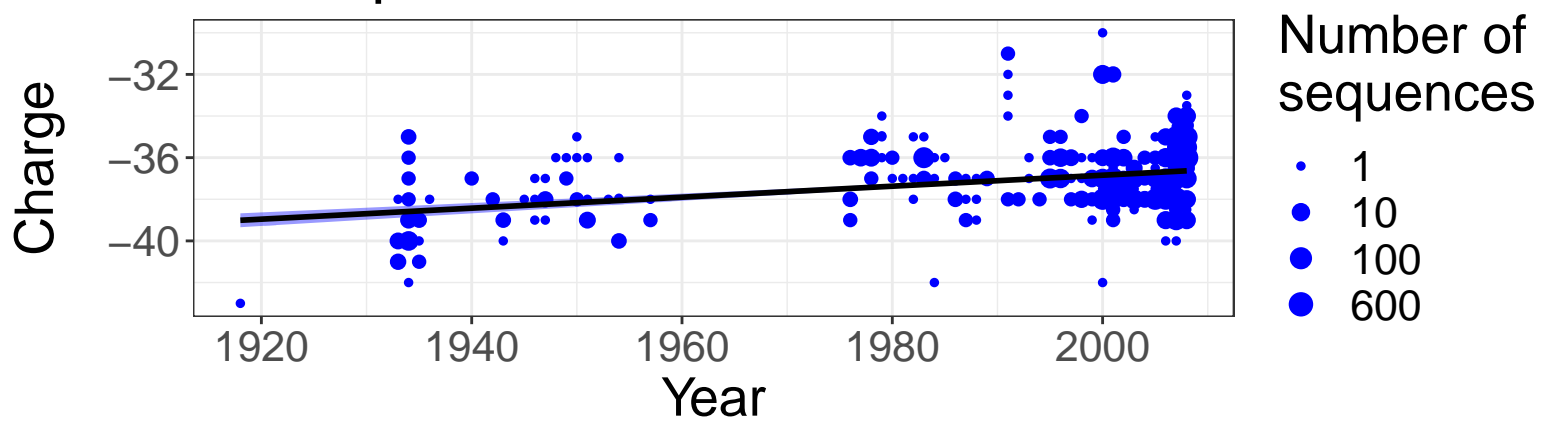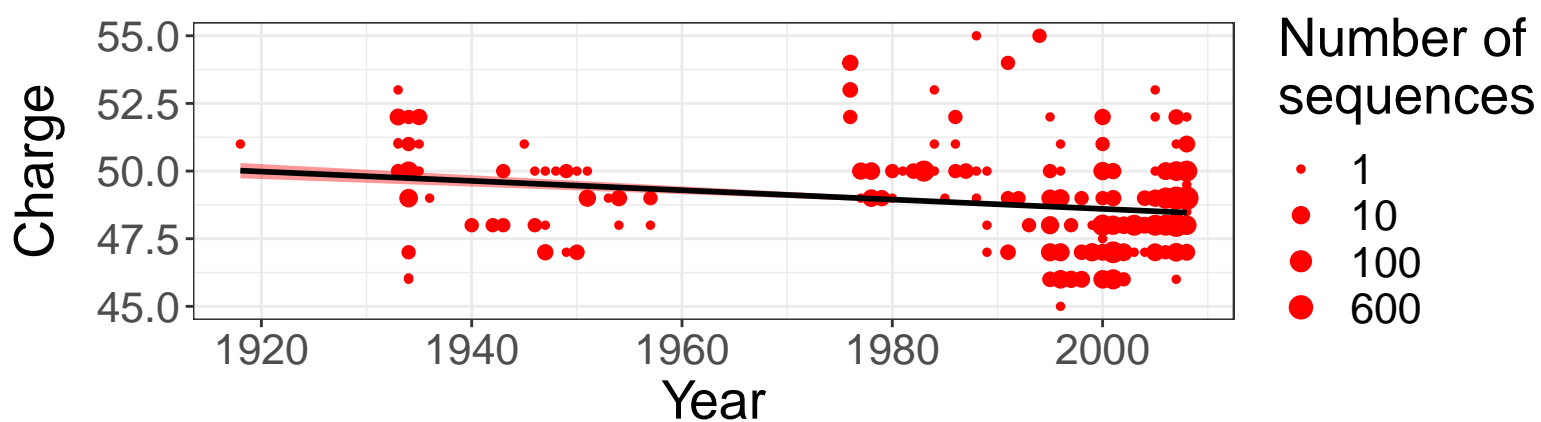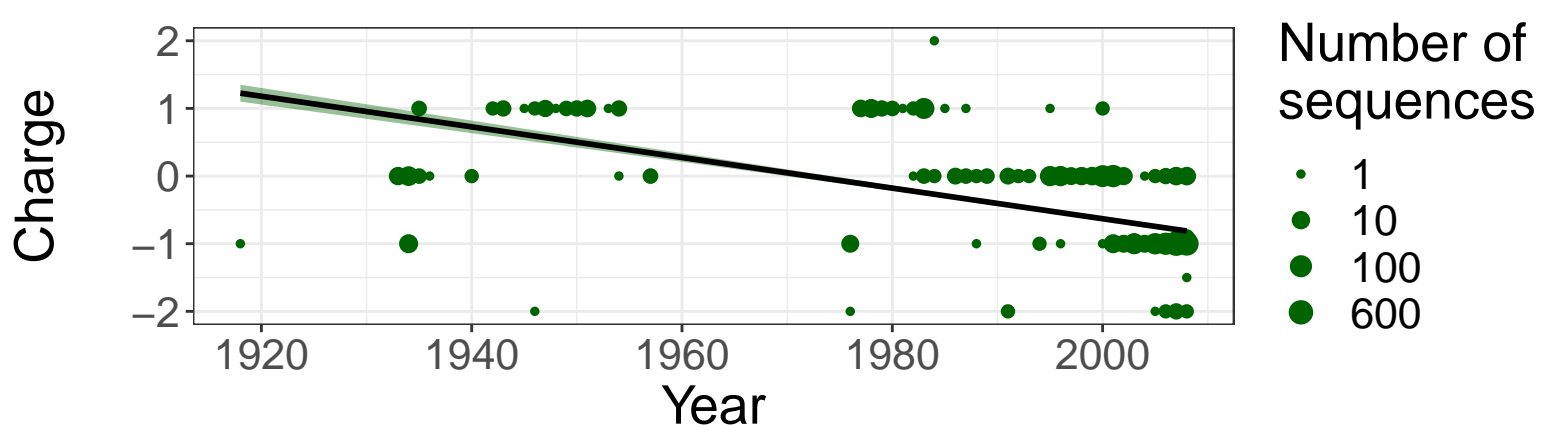**B** All sequences, HA1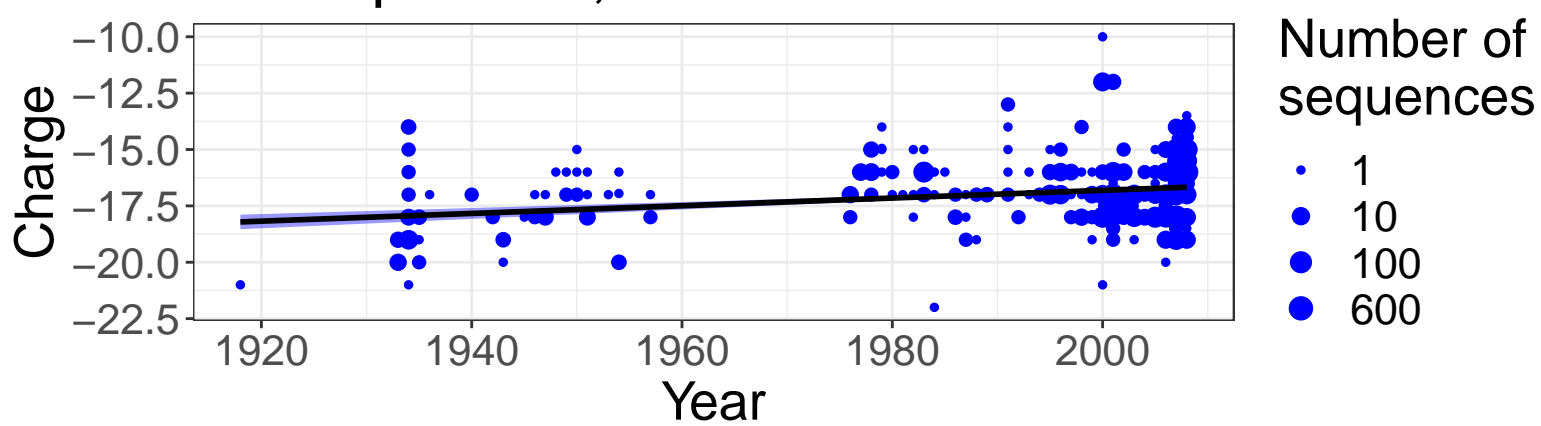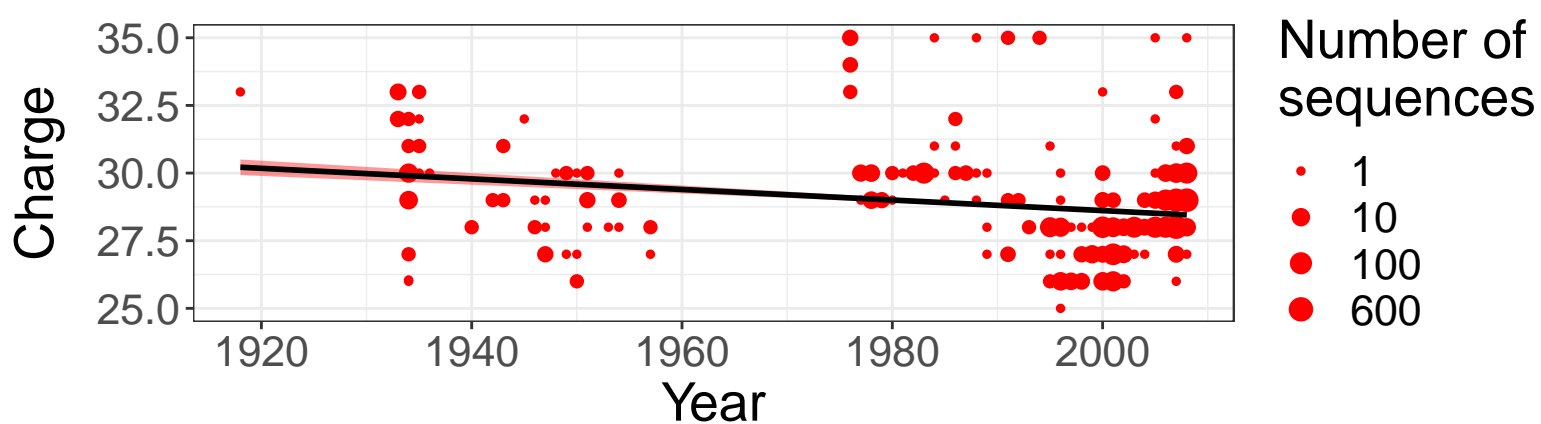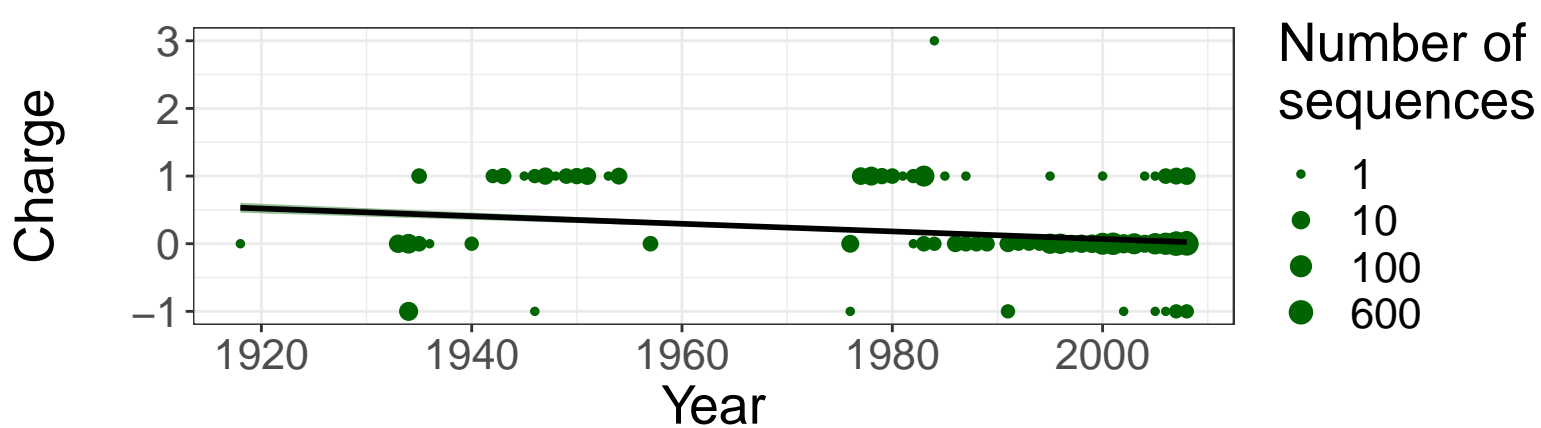**C** Yearly means, whole HA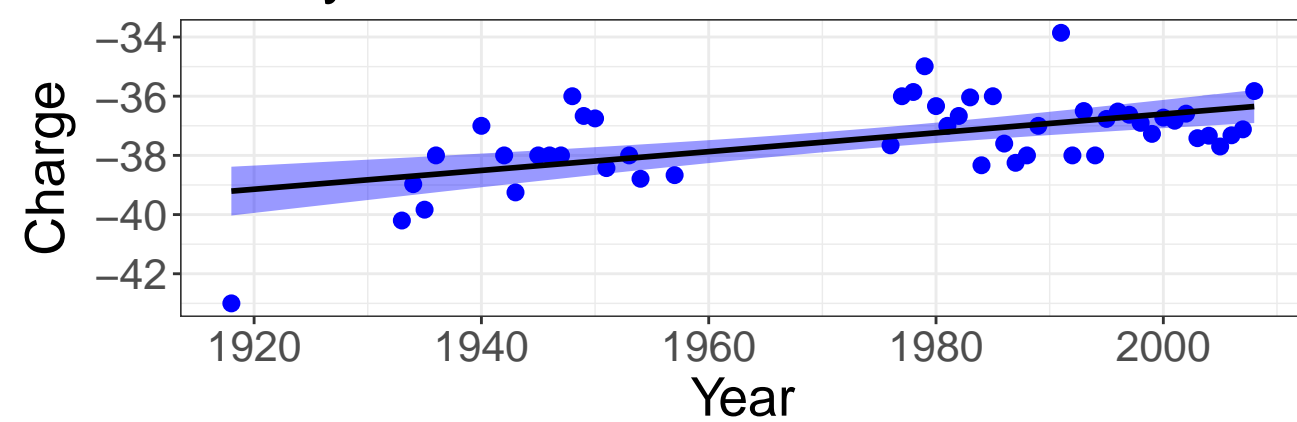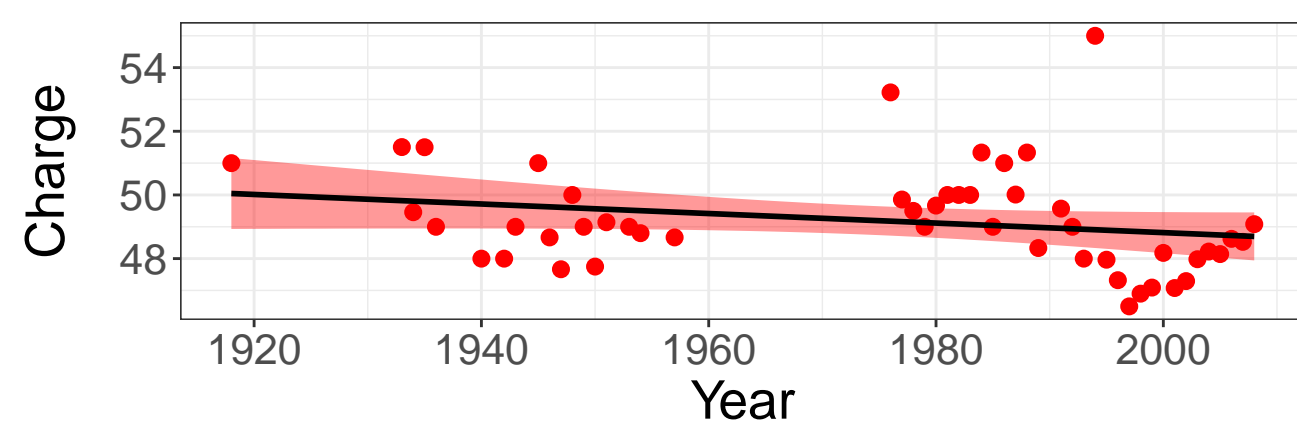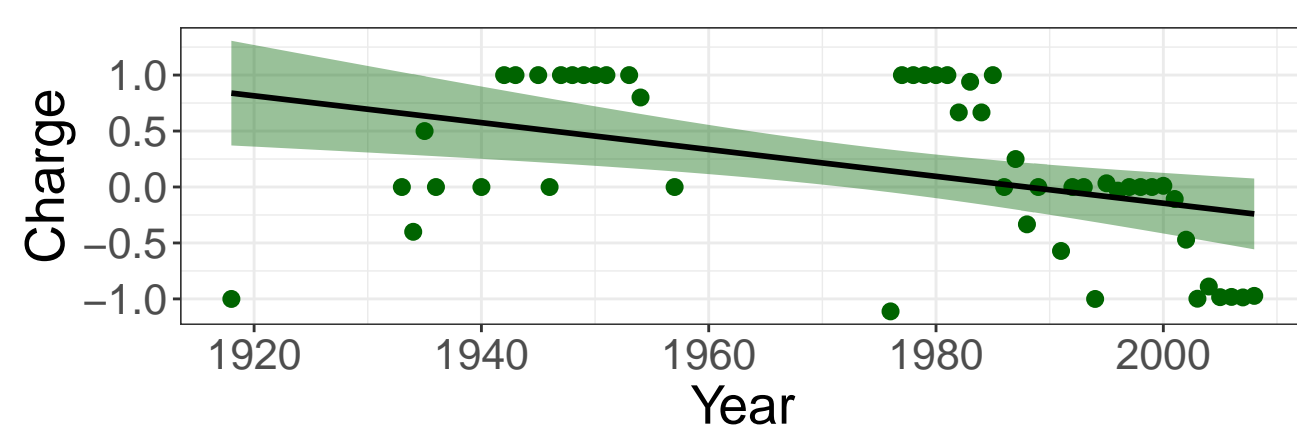**D** Yearly means, HA1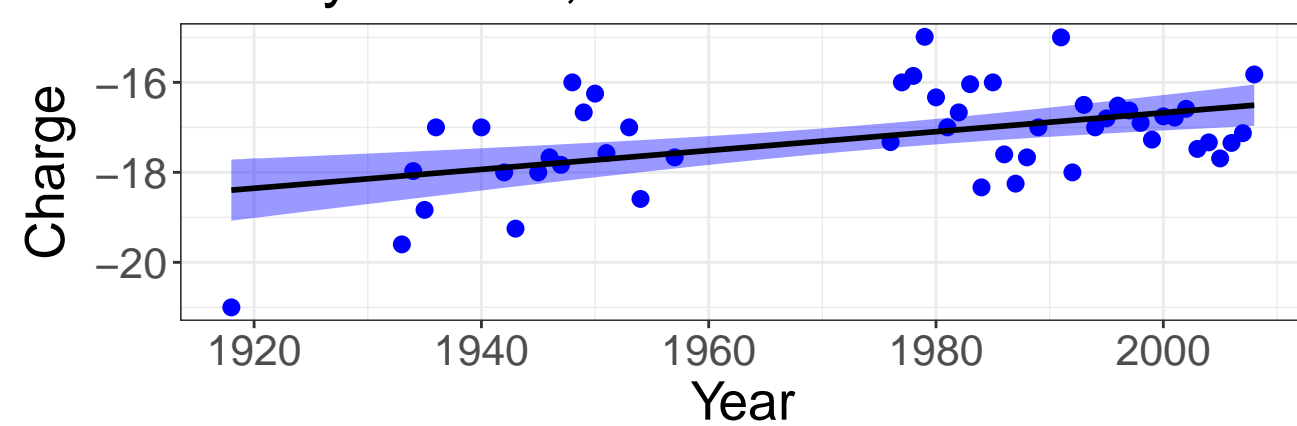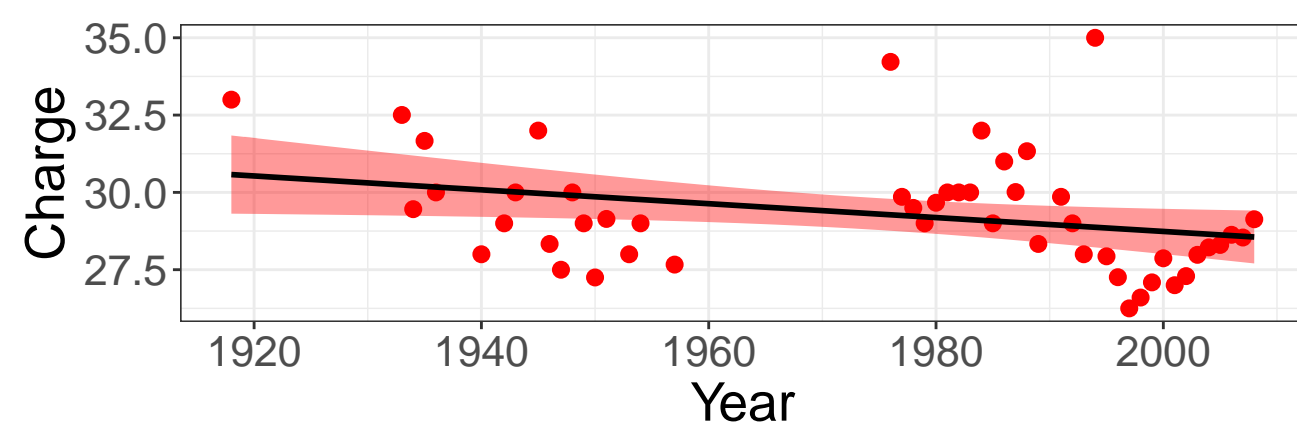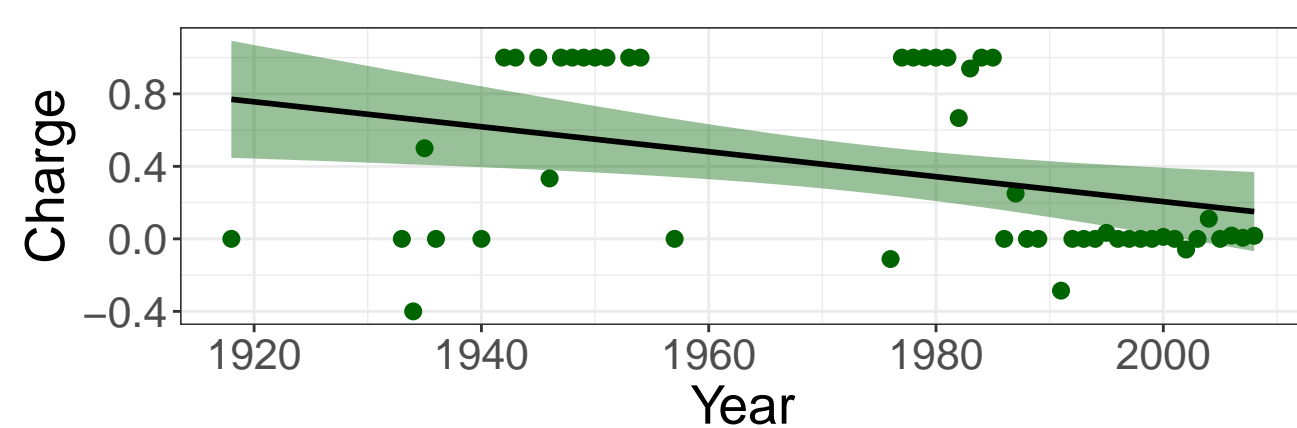

Supplement: S10 Fig — The red, blue, and green colours depict the positive, negative, and zero branches identified through hierarchical clustering with Ward’s method in S8 Fig. (A)–(B) Temporal trends by clusters with all sequences for (A) the whole HA and (B) HA1. (C)–(D) Yearly means for each cluster, for (A) the whole HA and (B) HA1. (PDF) [file pcbi.1007892.s011.pdf]

**A** whole HA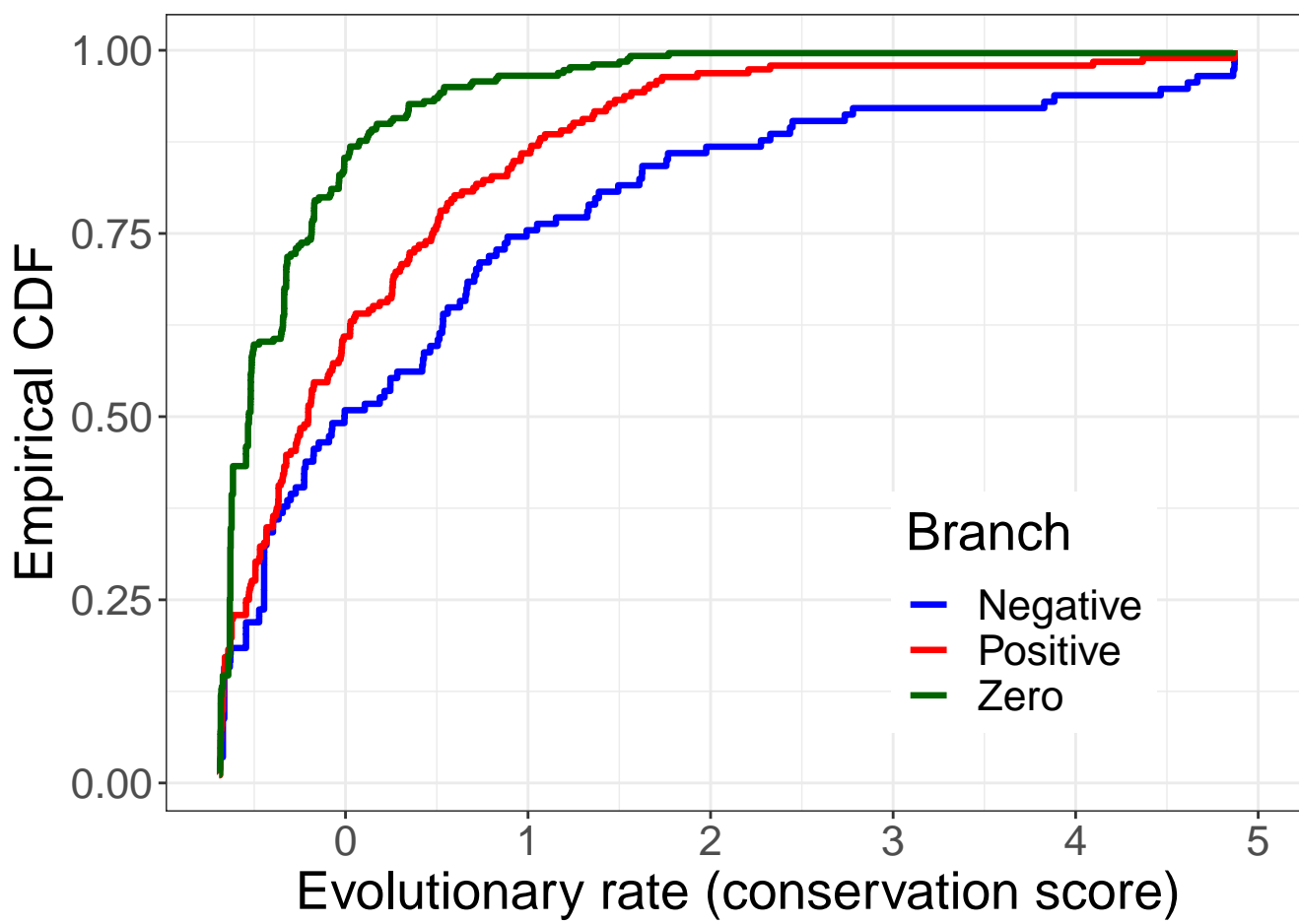**B** HA1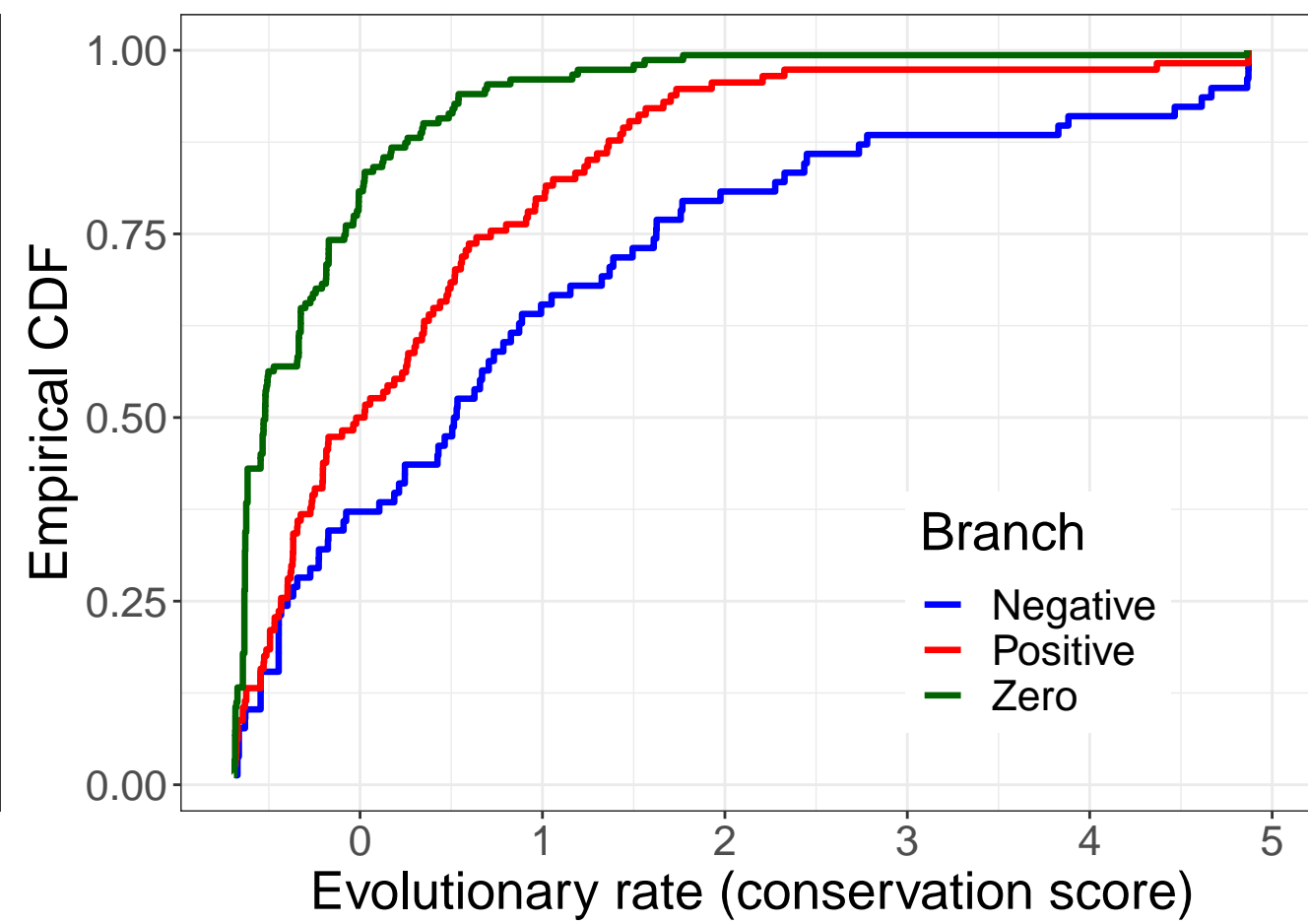**C** HA2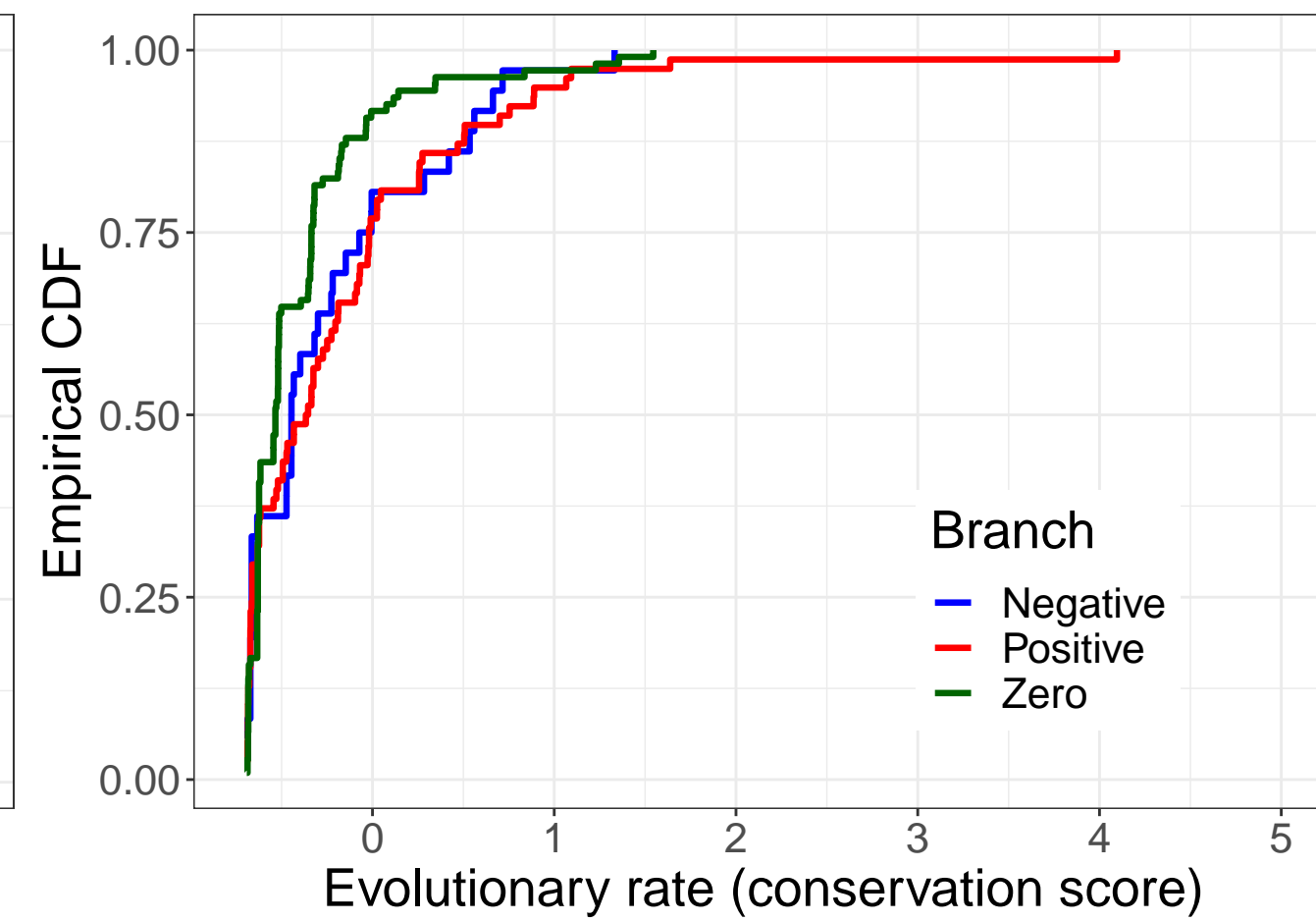**D** whole HA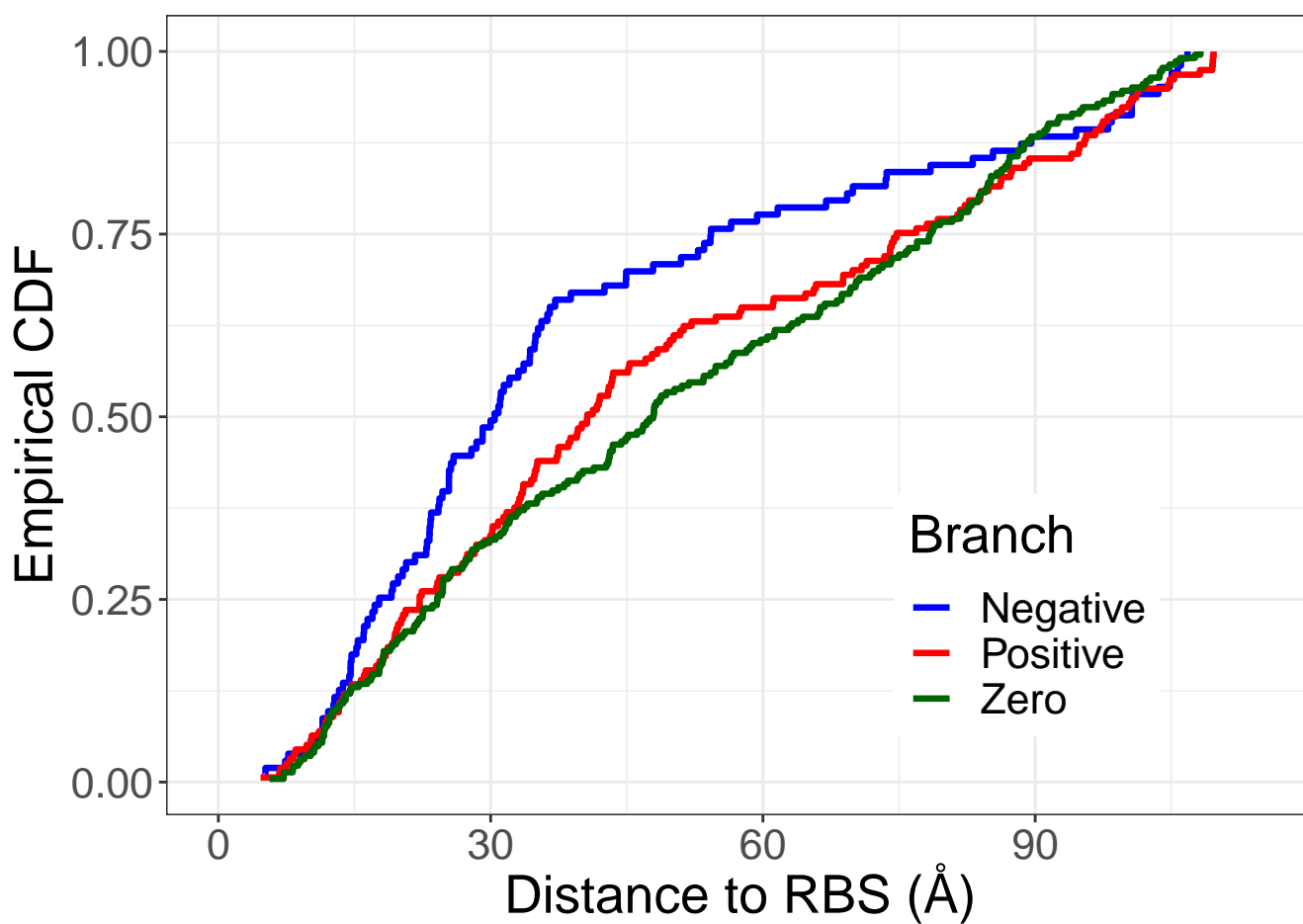**E** HA1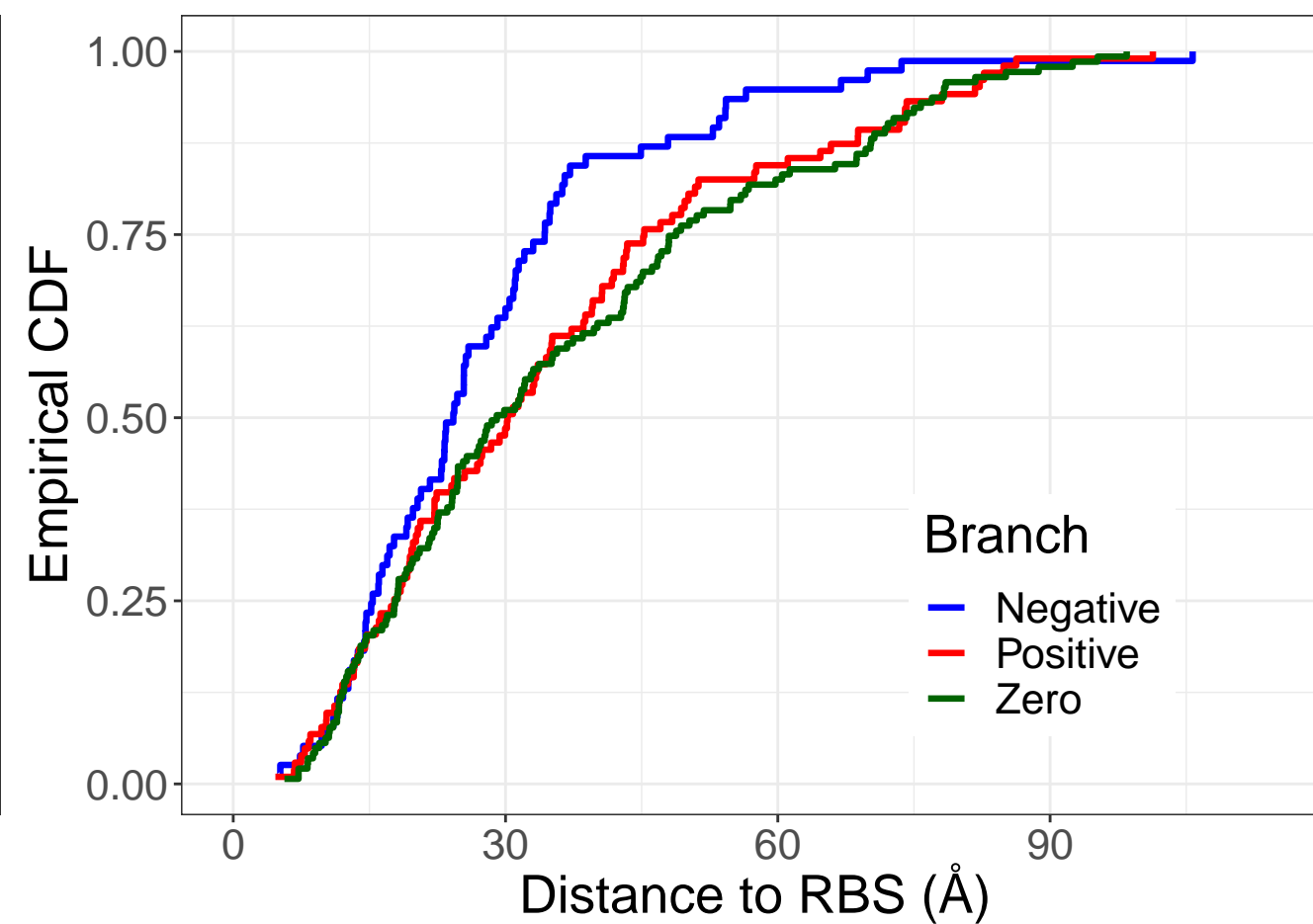**F** HA2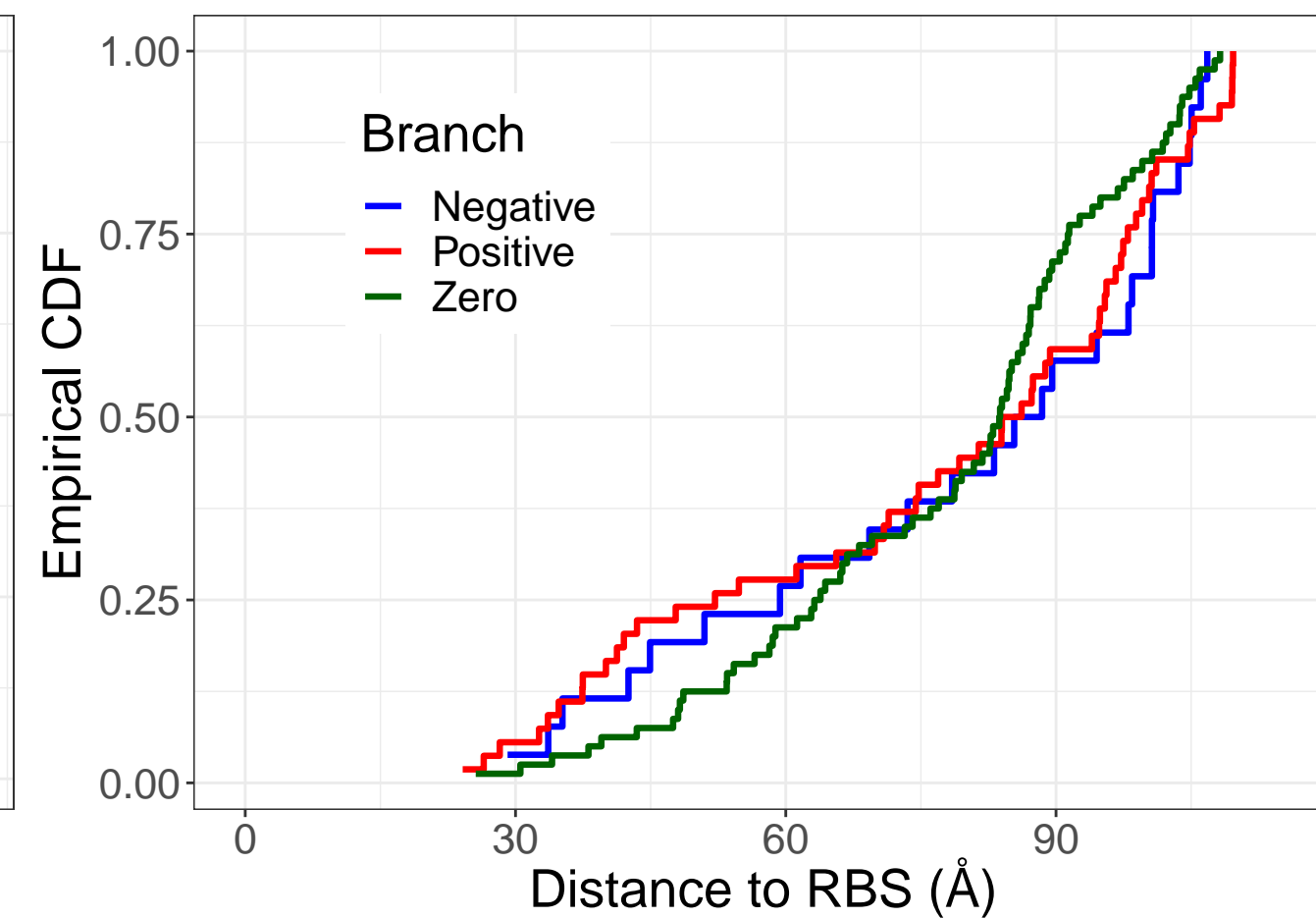**G** whole HA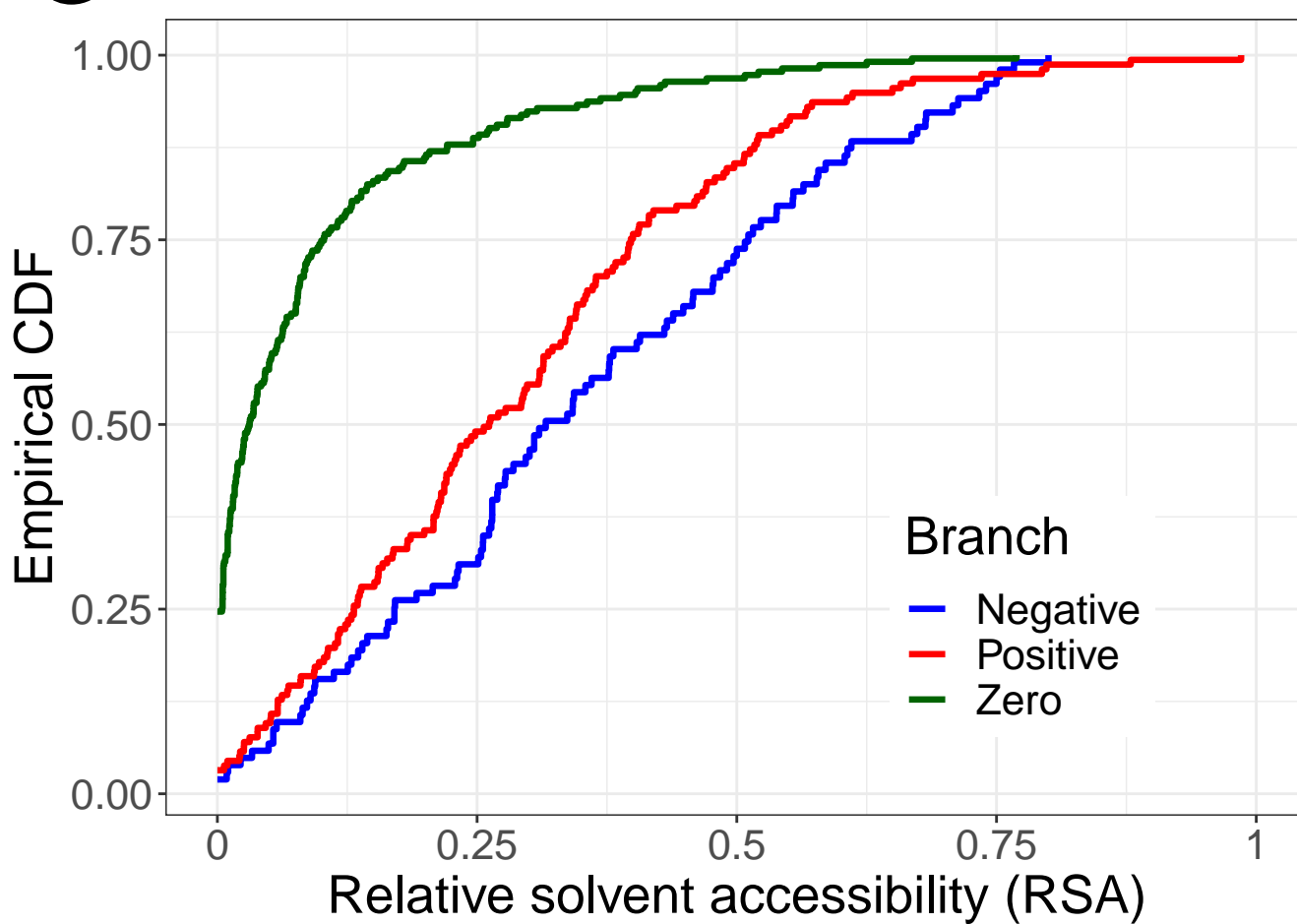**H** HA1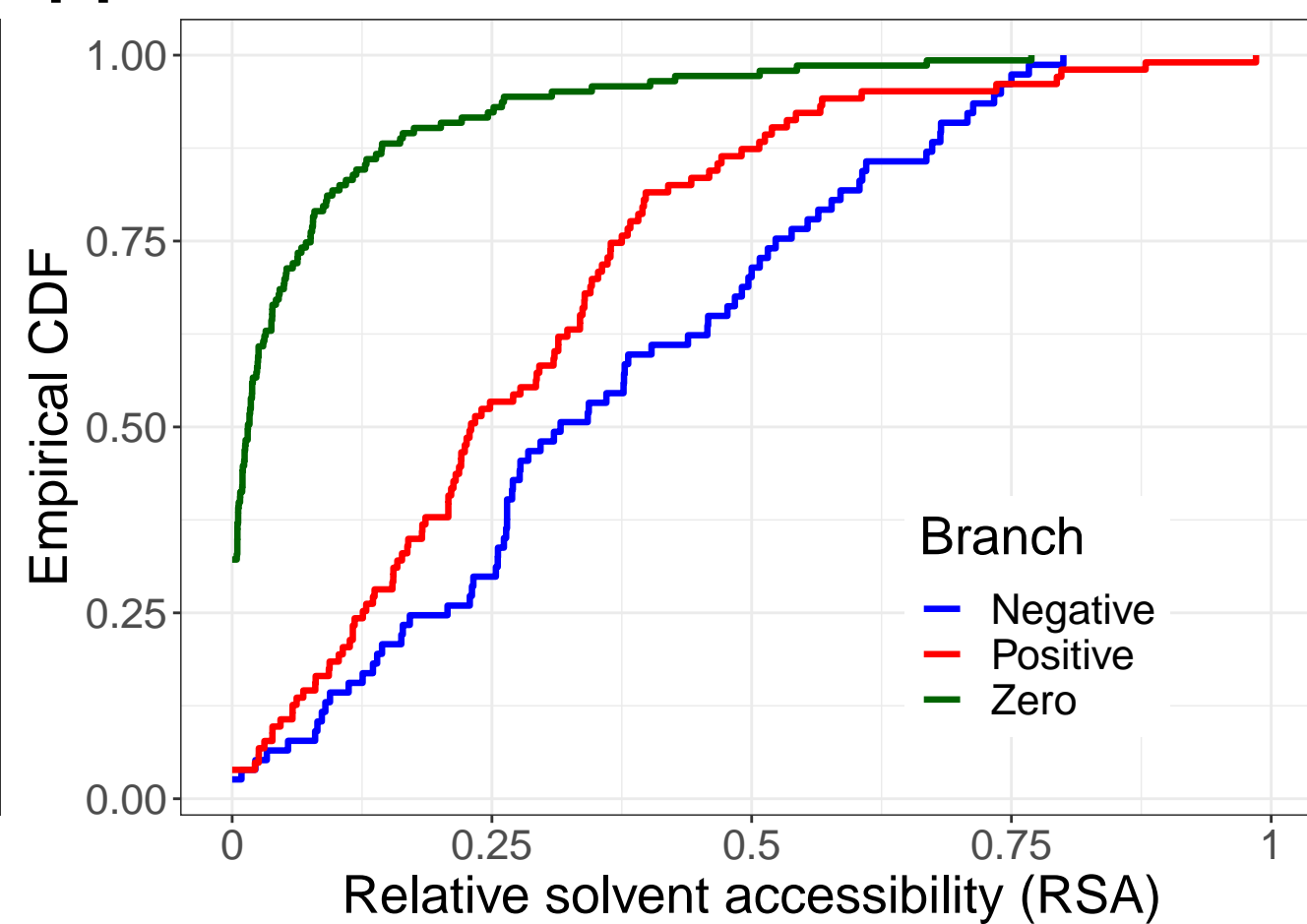**I** HA2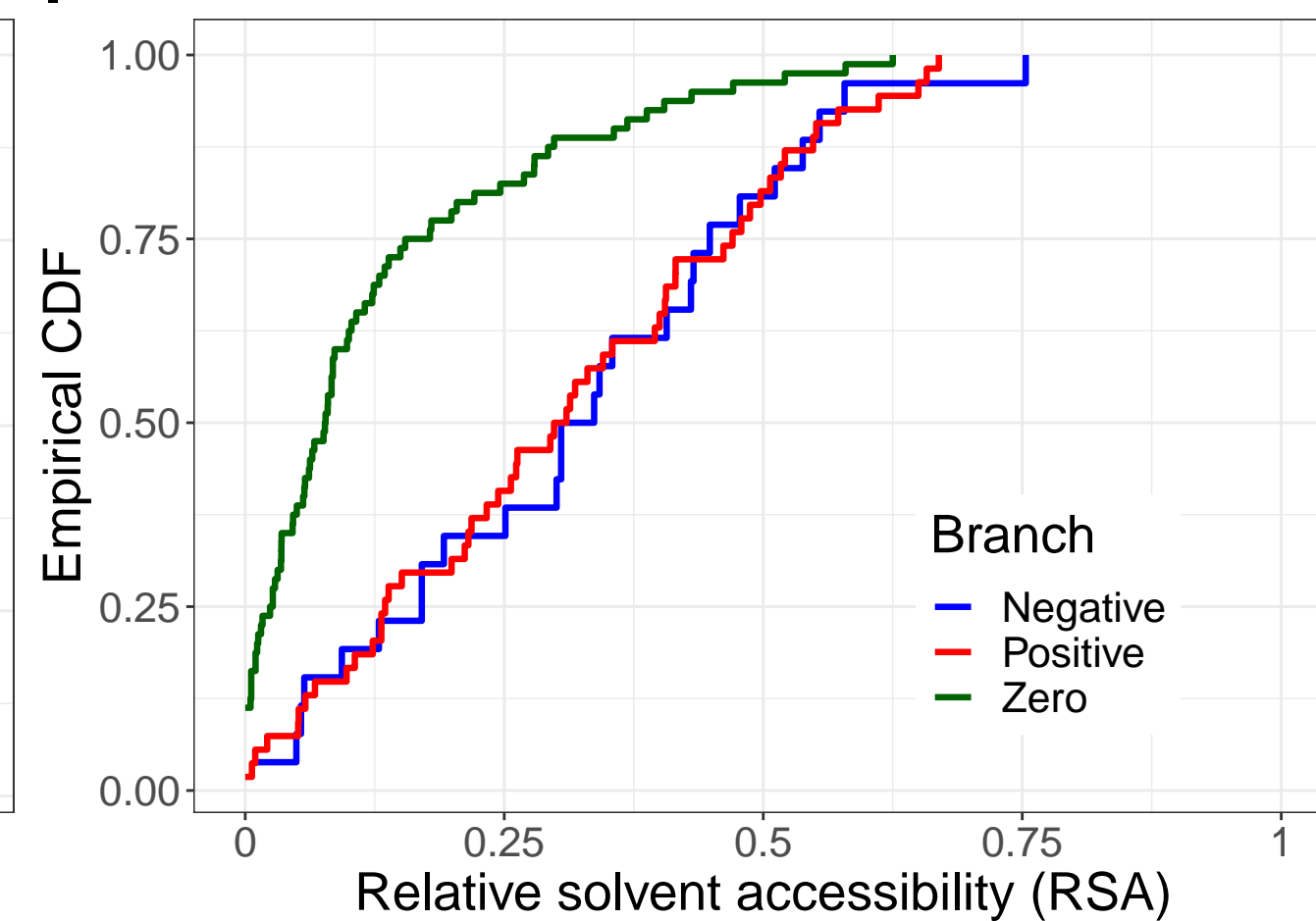

Supplement: S11 Fig — (A)–(C) are for distributions of evolutionary rates for (A) the whole HA, (B) HA1, and (C) HA2. (D)–(F) are for distributions of distances to the RBS for (D) the whole HA, (E) HA1, and (F) HA2. (G)–(I) are for distributions of RSAs for (G) the whole HA, (H) HA1, and (I) HA2. For the whole HA and RBS distributions, the positive-negative and negative-zero branches are significantly different (D = 0.2207, P = 0.003768 and D = 0.26109, P = 9.992 × 10^−5^, respectively), whereas the positive and zero branches are not significantly different (D = 0.10194, P = 0.2699). For RSA distributions, the positive-negative, positive-zero, and negative-zero branches are all significantly different (D = 0.17977, P = 0.03591; D = 0.57619, P < 2.2 × 10^−16^; and D = 0.62497, P < 2.2 × 10^−16^, respectively). For evolutionary rates, all the differences in branches are also significant (D = 0.17297, P = 0.02768 [positive-negative]; D = 0.32241, P = 2.215 × 10^−10^ [positive-zero]; and D = 0.38207, P = 1.837 × 10^−10^ [negative-zero]). The statistical significance of differences is similar for comparisons of branches with only HA1 sites, at a significance level of α = 0.05. (PDF) [file pcbi.1007892.s012.pdf]
