# Supplementary material for: Implications of localized charge for human influenza A H1N1 hemagglutinin evolution: Insights from deep mutational scans
Source: PLoS Comput Biol. 2020 Jun 25;16(6):e1007892. doi: 10.1371/journal.pcbi.1007892 (PMC7316228; doi:10.1371/journal.pcbi.1007892)
Supplement: S7 Fig — These values are plotted on the functional selection-expected value plot for HA residues on (A) a linear scale, and on (B) a log-scale. The probabilities of glycosylation were computed using DMS data and assuming independence of sites (see Materials and methods). (PDF) [file pcbi.1007892.s008.pdf]

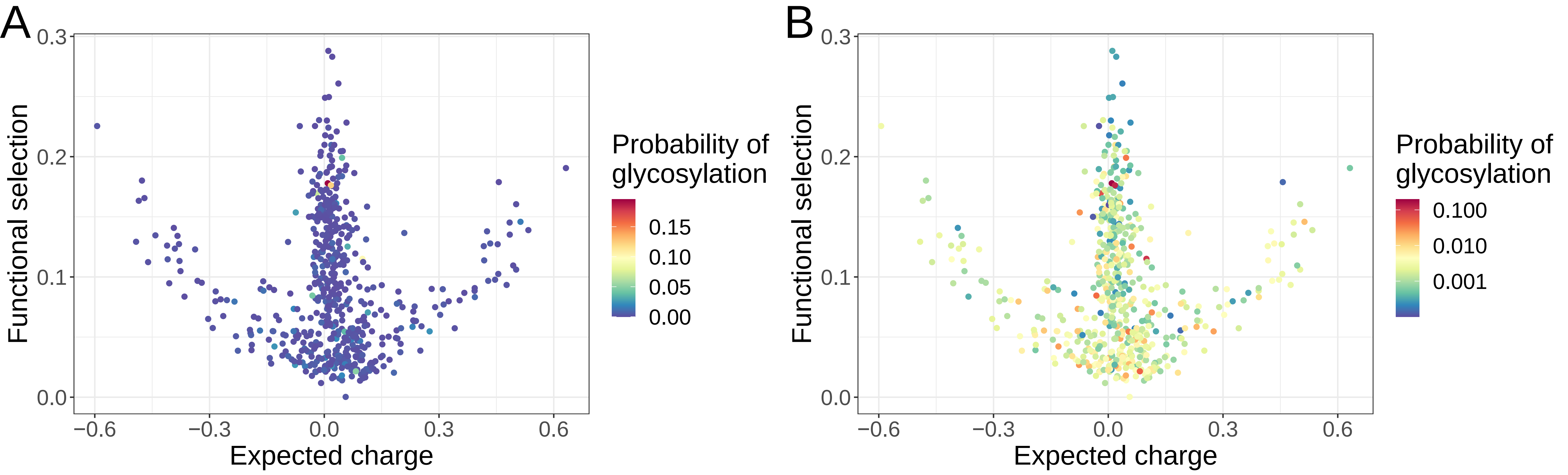
A
B
Functional selection
Expected charge
Probability of glycosylation
0.0
0.1
0.2
0.3
−0.6
−0.3
0.0
0.3
0.6
0.15
0.10
0.05
0.00
0.100
0.010
0.001
